# Effects of increased body mass index on employment status: a Mendelian randomisation study

**DOI:** 10.1038/s41366-021-00846-x

**Published:** 2021-06-22

**Authors:** Desmond D. Campbell, Michael Green, Neil Davies, Evangelia Demou, Joey Ward, Laura D. Howe, Sean Harrison, Keira J. A. Johnston, Rona J. Strawbridge, Frank Popham, Daniel J. Smith, Marcus R. Munafò, Srinivasa Vittal Katikireddi

**Affiliations:** 1 MRC/CSO Social & Public Health Sciences Unit, University of Glasgow, Glasgow, UK; 2 MRC Integrative Epidemiology Unit at the University of Bristol, Population Health Sciences, University of Bristol, Bristol, UK; 3 K.G. Jebsen Center for Genetic Epidemiology, Department of Public Health and Nursing, NTNU, Norwegian University of Science and Technology, Trondheim, Norway; 4 Mental Health & Wellbeing, University of Glasgow, Glasgow, UK; 5 Health Data Research, Glasgow, UK; 6 Cardiovascular Medicine, Department of Medicine Solna, Karolinska Institutet, Stockholm, Sweden; 7 School of Psychological Science, University of Bristol, Bristol, UK

**Keywords:** Obesity, Epidemiology

## Abstract

**Background:**

The obesity epidemic may have substantial implications for the global workforce, including causal effects on employment, but clear evidence is lacking. Obesity may prevent people from being in paid work through poor health or through social discrimination. We studied genetic variants robustly associated with body mass index (BMI) to investigate its causal effects on employment.

**Dataset/methods:**

White UK ethnicity participants of working age (men 40–64 years, women 40–59 years), with suitable genetic data were selected in the UK Biobank study (*N* = 230,791). Employment status was categorised in two ways: first, contrasting being in paid employment with any other status; and second, contrasting being in paid employment with sickness/disability, unemployment, early retirement and caring for home/family. Socioeconomic indicators also investigated were hours worked, household income, educational attainment and Townsend deprivation index (TDI). We conducted observational and two-sample Mendelian randomisation (MR) analyses to investigate the effect of increased BMI on employment-related outcomes.

**Results:**

Regressions showed BMI associated with all the employment-related outcomes investigated. MR analyses provided evidence for higher BMI causing increased risk of sickness/disability (OR 1.08, 95% CI 1.04, 1.11, per 1 Kg/m^2^ BMI increase) and decreased caring for home/family (OR 0.96, 95% CI 0.93, 0.99), higher TDI (Beta 0.038, 95% CI 0.018, 0.059), and lower household income (OR 0.98, 95% CI 0.96, 0.99). In contrast, MR provided evidence for no causal effect of BMI on unemployment, early retirement, non-employment, hours worked or educational attainment. There was little evidence for causal effects differing by sex or age. Robustness tests yielded consistent results.

**Discussion:**

BMI appears to exert a causal effect on employment status, largely by affecting an individual’s health rather than through increased unemployment arising from social discrimination. The obesity epidemic may be contributing to increased worklessness and therefore could impose a substantial societal burden.

## Introduction

Obesity has increased markedly over the last few decades throughout the world. In 2015 high BMI accounted for 4 million deaths globally, nearly 40% of which occurred in non-obese people [1]. This obesity epidemic has been underpinned by a mean shift in the population distribution of body mass index (BMI) rather than just an increase in the high BMI tail of the distribution. While there has been a considerable focus on the associated healthcare costs arising from higher weight [2], the broader adverse consequences to society are less understood [3]. However, these impacts may be substantial, with the broader economic costs potentially dwarfing the direct healthcare costs. The impacts of poor health on work are of substantial policy interest and provide an important rationale for government action [4].

Employment status is a measure of an individual’s economic activity, including being employed (in paid work), unemployed (not employed but capable of paid work), incapacity benefit (not able to work due to poor health), being a carer (not in paid work to allow looking after someone else) and retired. Health and health inequalities have been found to be related to employment measures [5–7]. However, the direction of causation has been less thoroughly explored. The pathways by which BMI impacts on employment might be varied. Some of these effects may be directly related to poor health whereas others may be related to reduced employability even if health is unaffected for example, due to workplace discrimination. Obese individuals face inequalities in hiring, wages, promotions, job termination and negative attitudes from co-workers [8]. For instance, studies have found obesity negatively impacting on perceived job suitability [9]. In contrast, some adverse impacts are thought to arise specifically as a consequence of poor health, with obesity linked to greater sickness absence due to illness, injury and disability [10].

Despite policy interest in the adverse economic and employment impacts of obesity, establishing causality is challenging. The direction of the causal relationship may be unclear, since it is possible for changes in employment to cause weight change. As in many areas of research, randomised trials are infeasible and unethical. Furthermore, traditional epidemiological studies are susceptible to confounding due to common factors being a cause of both weight and employment outcomes. These confounders can be particularly challenging to address given the difficulty of accurately measuring socioeconomic variables across the life course. In addition, reverse causation can occur, for instance employment could affect weight.

Mendelian randomisation (MR) can be considered an instrumental variable approach, which uses genetic variants as instruments to allow assessment of causal effects using observational data [11–13]. By studying genetic variants that are correlated with the exposure of interest (in this case, BMI), concerns regarding reverse causation are mitigated since an individual’s genotype is established at conception. MR studies are also much less susceptible to confounding than traditional observational study designs. In other words, MR studies can be thought of as akin to a randomised trial where the exposure of interest is allocated at conception. They therefore estimate the causal effects of a lifelong tendency to an exposure, rather than the short-term effects at a specific point in time. MR studies are subject to some important assumptions for estimating causal effects without bias—most notably, the genetic variants should only affect the outcome (employment status) through the exposure (BMI). In MR, this assumption may be violated if the genetic variants have multiple functions, referred to as horizontal pleiotropy.

To investigate the causal effects of BMI on employment status, we assessed the relationship between genetic variants robustly associated with BMI on employment in the UK Biobank study. Furthermore, we investigated the impact of BMI on indicators related to socioeconomic conditions—namely, household income, educational attainment and area-based deprivation.

## Methods

### Study population

The UK Biobank study collected data on half a million individuals aged between 40 and 69 from across mainland Britain (fieldwork conducted 2006–10) [14]. Participants were excluded from the current study if they (1) were not of working age (i.e., above retirement age at the time of assessment—60 years for women, 65 years for men); (2) self-reported ethnicity other than White UK; (3) had withdrawn from the study (before 26 Jan 2020); (4) were overly genetically related; (5) had other issues with the validity of their genetic data; or (6) did not have a value for one of the outcomes investigated. Participant exclusion is detailed in Appendix 2.1 and in a STROBE flowchart (Fig. S1).

### Genetic variants associated with BMI

We identified genetic instruments for BMI based on a set of single nucleotide polymorphisms (SNPs) which were robustly associated in a genome wide association study (GWAS) [15]. This is the most recent GWAS to our knowledge that omits UK Biobank which if included would introduce sample overlap bias. We rejected associations not reaching genome wide significance (*P* value ≤ 5 × 10^−8^). SNPs were then rejected based on Hardy Weinberg Equilibrium, Information Content and Minor Allele Frequency criteria. After clumping of BMI associating SNPs, this yielded a subset of 77 independent SNPs (Table S1). SNP exclusions are detailed in Appendix 2.3 and in a flowchart (Fig. S2). Searches by Locke et al. [15] (and Speliotes et al. [16]) did not find associations between these SNPs and characteristics unrelated to BMI that might directly affect employment outcomes (such as intelligence), i.e. they did not find evidence for horizontal pleiotropy for these SNPs.

We generated an unweighted polygenic score for BMI for each subject (use of an unweighted score is justified in Appendix 2.4). This was calculated as the number of risk alleles the subject carried across all instrument SNPs. To ensure the directions of association were consistent for each estimate, variants were harmonised using the TwoSampleMR package within R [17]. We confirmed the relationship between BMI and the instrument SNPs in the study sample by regressing BMI on the polygenic risk score with adjustment for age, sex, study assessment centre, and 40 genetic principal components (GPCs) (see Appendix for details). Inclusion of GPCs as covariates in a regression is a standard way of correcting for confounding between genetic regressors and the outcome (such confounding is also known as population stratification). We chose to use all 40 GPCs, available from UK Biobank, as covariates. Although some of these may not be significant regressors, their inclusion as covariates does no harm other than potentially reducing power. We favoured controlling for population stratification over power.

### Exposure and outcomes

Participant BMI was calculated from their height and weight measured during their initial UK Biobank Assessment. Current employment status was self-reported, with the five most common categories being: employed, retired, sickness/disability (i.e., not working due to health), caring for home/family, and unemployed. As the analysis sample only includes those of working age anyone retired is in early retirement. These were recoded into four binary variables comparing each other category against employment. We also encoded another binary variable contrasting all other categories (hereafter referred to as non-employment) against paid employment. Respondents could endorse multiple categories (<8% participants). In such cases when coding the binary variables, ‘employed’ took priority over the contrasted employment category. We also considered self-reported weekly hours in paid employment, Townsend deprivation index (TDI), household income and highest educational attainment as outcomes. TDI is a measure of area-based deprivation, based on the levels of unemployment, non-car ownership, non-home ownership and household overcrowding within small census areas [18]. A greater TDI score implies a greater deprivation. Highest educational attainment was coded as an ordinal variable with ordered levels: (1) CSEs or equivalent, (2) O levels/GCSEs or equivalent, (3) A levels/AS levels or equivalent, (4) NVQ or HND or HNC or equivalent, (5) other professional qualifications, e.g. nursing, teaching, (6) college or university degree. Household income was coded as an ordinal variable with ordered levels: (1) Less than 18,000, (2) 18,000–30,999, (3) 31,000–51,999, (4) 52,000–100,000, (5) greater than 100,000 GBP per annum. Table S2 lists the UK Biobank fields used for construction of the study variables.

### Statistical analyses

We first investigated the observational association between BMI and each employment outcome of interest for comparison purposes. Regression models were estimated with adjustment for age (as a continuous variable), sex, study assessment centre, and 40 GPCs. We decided against including additional covariates as many variables which were collected at baseline could be considered likely mediators, rather than confounders.

To assess potential causal relationships, we conducted a two-sample MR analysis using the SNP-exposure associations reported by Locke et al. [15]. and the SNP-outcome associations from the study sample. This was implemented using the R package TwoSampleMR [17]. The association between the genetic instrument SNPs and the outcomes were assessed by running a series of regressions of each employment outcome on each instrument SNP, adjusting for age, sex, study assessment centre and GPCs (implemented using PLINK 1.9 [19]). For binary outcomes, regressions were logistic, for continuous outcomes, regressions were linear and for ordinal outcomes, regressions were ordinal.

To assess the potential for violations of the MR assumptions, we estimated causal effects using the wide range of MR estimators available in the TwoSampleMR R package [17]. Heterogeneity in causal effect estimates from the set of instrument SNPs was assessed with Cochran’s Q (assuming balanced pleiotropy) and Rücker’s Q (assuming unbalanced pleiotropy) using the RadialMR R package [20]. Using these as inputs, we applied the Rücker model selection framework to identify the best fitting model between fixed effect and random effect versions of the IVW and Egger methods [21]. We conducted unbalanced pleiotropy tests. We also calculated $I_{GX}^2$, a measure of the degree of violation of the No Measurement Error assumption for SNP-exposure associations [22]. In order to assess whether any single SNP was driving effect estimates we conducted single SNP MR analyses and leave one SNP out MR analyses. Analyses were repeated with outlier SNPs (identified via RadialMR [20]) excluded, but this had little impact on the results. Further details are provided in Appendix 2.8.

Given we expected differences in the impact of BMI between males and females, the two-sample MR analyses were repeated stratified by sex. Wald tests were used to compare effect estimates between males and females. We used the same instrument SNP set for the sex-stratified MR analyses as for the main MR analyses. Justification for this along with further details of sex difference testing is provided in Appendix 2.9. Similarly, we tested whether there was evidence for a moderating effect of age on the MR estimates. We repeated the MR analyses stratified by age band. An F test was used to test whether MR estimate varied with age band. For further details see Appendix 2.10.

## Results

The analytical sample comprised 230,791 genetically unrelated participants of White UK ethnic origin of working age. Table 1 shows descriptive information for the sample. The study population included more men (54.5%) than women, and being in paid work was the most common employment status (72% of men and 79% of women). Men in the sample tended to be older, worked more hours weekly and reported early retirement more frequently.

**Table 1:** Descriptive information for the analytical sample.

	Female	Male	Overall
**Sample size**	104945	125846	230791
**Age (mean (SD))**	50.96 (5.55)	54.46 (7.04)	52.87 (6.64)
**BMI (mean (SD))**	26.82 (5.32)	27.84 (4.30)	27.38 (4.82)
**Employment Category (%)**			
**Employed**	83292 (79.4%)	90303 (71.8%)	173595 (75.2%)
**Retired Early**	6768 (6.4%)	24036 (19.1%)	30804 (13.3%)
**Sick/Disabled**	4978 (4.7%)	6848 (5.4%)	11826 (5.1%)
**Family/Carer**	7534 (7.2%)	1080 (0.9%)	8614 (3.7%)
**Unemployed**	1640 (1.6%)	3558 (2.8%)	5198 (2.3%)
**Not in Paid Work**	20791 (19.8%)	34507 (27.4%)	55298 (24.0%)
**Hours Worked Weekly (mean (SD))**	32.28 (11.60)	40.34 (11.07)	36.47 (12.02)
**Townsend Deprivation Index (mean (SD))**	−1.49 (2.93)	−1.48 (3.02)	−1.48 (2.98)
**Household Income (%)**			
**Less than 18,000**	13707 (13.1%)	18055 (14.3%)	31762 (13.8%)
**18,000 to 30,999**	19968 (19.0%)	25266 (20.1%)	45234 (19.6%)
**31,000 to 51,999**	28012 (26.7%)	33279 (26.4%)	61291 (26.6%)
**52,000 to 100,000**	24485 (23.3%)	29693 (23.6%)	54178 (23.5%)
**Greater than 100,000**	6303 (6.0%)	8030 (6.4%)	14333 (6.2%)
**NA**	12470 (11.9%)	11523 (9.2%)	23993 (10.4%)
**Highest Educational Attainment (%)**			
**None of the below**	9321 (8.9%)	17023 (13.5%)	26344 (11.4%)
**CSEs or equivalent**	5709 (5.4%)	5035 (4.0%)	10744 (4.7%)
**O levels/GCSEs or equivalent**	15486 (14.8%)	12937 (10.3%)	28423 (12.3%)
**A levels/AS levels or equivalent**	6896 (6.6%)	6562 (5.2%)	13458 (5.8%)
**NVQ or HND or HNC or equivalent**	13834 (13.2%)	20850 (16.6%)	34684 (15.0%)
**Other professional qualifications eg: nursing, teaching**	15518 (14.8%)	18354 (14.6%)	33872 (14.7%)
**College or University degree**	37508 (35.7%)	44076 (35.0%)	81584 (35.3%)
**NA**	673 (0.6%)	1009 (0.8%)	1682 (0.7%)

Results for the regression of employment-related outcomes on BMI (observational associations) are presented in Table 2, with results for covariates shown in Table S3. In comparison to the reference group of employed people, higher BMI was associated with greater odds of not being in paid employment (OR per 1 Kg/m^2^ 1.015, 95% CI: 1.013, 1.017). Higher BMI was associated with greater odds of being sick/disabled (OR 1.082, 95% CI: 1.078, 1.086), or unemployed (OR 1.029, 95% CI: 1.023, 1.035), but reduced odds of caring for home/family (OR 0.992, 95% CI: 0.988, 0.997) or being retired (OR 0.994, 95% CI: 0.990, 0.997). Higher BMI was associated with higher weekly hours in paid employment (Hours per 1 Kg/m^2^ 0.179, 95% CI: 0.168, 0.190), and higher (more deprived) TDI (TDI per 1 Kg/m^2^ 0.056, 95% CI: 0.053, 0.058). Higher BMI also associated with lower household income level (OR 0.974, 95% CI: 0.972, 0.975), and lower maximum education level (OR 0.957, 95% CI: 0.956, 0.959). For the ordinal outcomes (household income level and maximum education level) the estimate given is the proportional change in odds for any chosen category upon a 1 Kg/m^2^ increase in BMI.

**Table 2:** Results for the regression of employment related outcomes on BMI.

Employment Category	Odds Ratio	Odds Ratio 95% CI	Odds Ratio P Value	N of Complete Obs
Not in paid employment	1.015	(1.013, 1.017)	6.5E-43	228217
Sick/Disabled	1.082	(1.078, 1.086)	<1.0E-300	184873
Caring for Home/Family	0.992	(0.9878, 0.9967)	7.2E-04	181883
Retired	0.994	(0.9903, 0.9968)	1.0E-04	203987
Unemployed	1.029	(1.023, 1.035)	3.8E-22	178466
**Outcome**	**Beta**	**Beta 95% CI**	**Beta P Value**	**N of Complete Obs**
Townsend Deprivation Index	0.056	(0.05327, 0.05802)	<1.0E-300	229790
Hours Worked	0.179	(0.1675, 0.1904)	5.7E-206	171316
**Outcome**	**Odds Ratio**	**Odds Ratio 95% CI**	**Odds Ratio P Value**	**N of Complete Obs**
Highest Educational Attainment	0.957	(0.9557, 0.9586)	<1.0E-300	228365
Household Income	0.974	(0.9722, 0.9754)	6.3E-215	205970

Effects for all employment categories and outcomes are adjusted for age, sex, study assessment centre, and genetic principal components.

Household Income is additionally adjusted for number in household.

The unweighted polygenic risk score had an adjusted partial R^2^ of 1.5% (i.e., explained 1.5% of the variance in BMI in the sample). This was highly significant confirming that the instrument SNP set associated with the exposure of interest (Table S4). A unit increase in the polygenic risk score predicted a 0.11 increase in BMI, which is similar in magnitude to that of a polygenic score reported by Locke et al. [15]. F-statistics from the Locke et al. regression of BMI on individual SNPs ranged from 30.1 to 262, with a median of 40.5. Thus the rule of thumb weak instruments criterion of F <10 was exceeded in all cases, suggesting minimal risk of weak instrument bias [23]. The F statistic associated with our polygenic risk score was 3709.

**Fig. 1:**
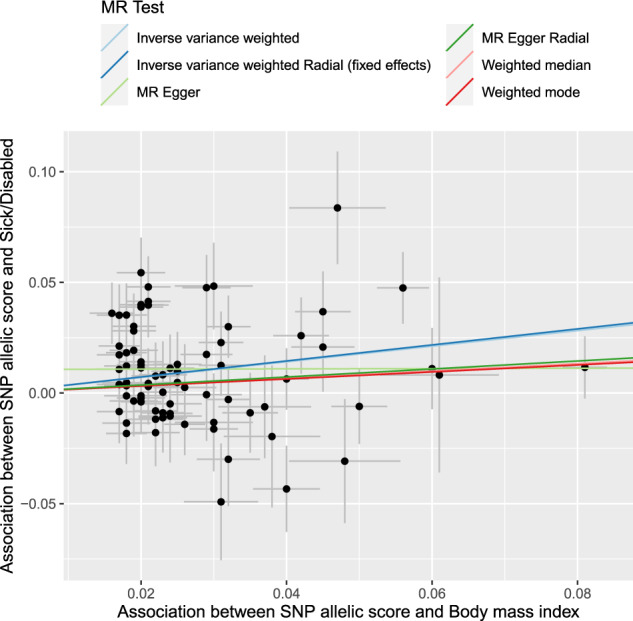
Scatter plot of sick/disabled-SNP associations versus exposure-SNP associations. BMI body mass index, SNP single nucleotide polymorphism. x-axis—BMI-SNP regression coefficient estimates from Locke et al. (normalised BMI), y-axis—Sick/Disabled-SNP log odds from UK Biobank regressions. Also plotted are the fits for several causal effect estimation methods.

Figure 1 shows a scatter plot of the SNP-outcome against SNP-exposure relationship for the sick/disabled outcome. Consistent with the assumptions of MR analyses, SNPs more strongly associated with the exposure, were also more strongly associated with the outcome. For similar scatter plots of the SNP-outcome against SNP-exposure relationships for the other outcomes, see Appendix 3.5.

**Fig. 2:**
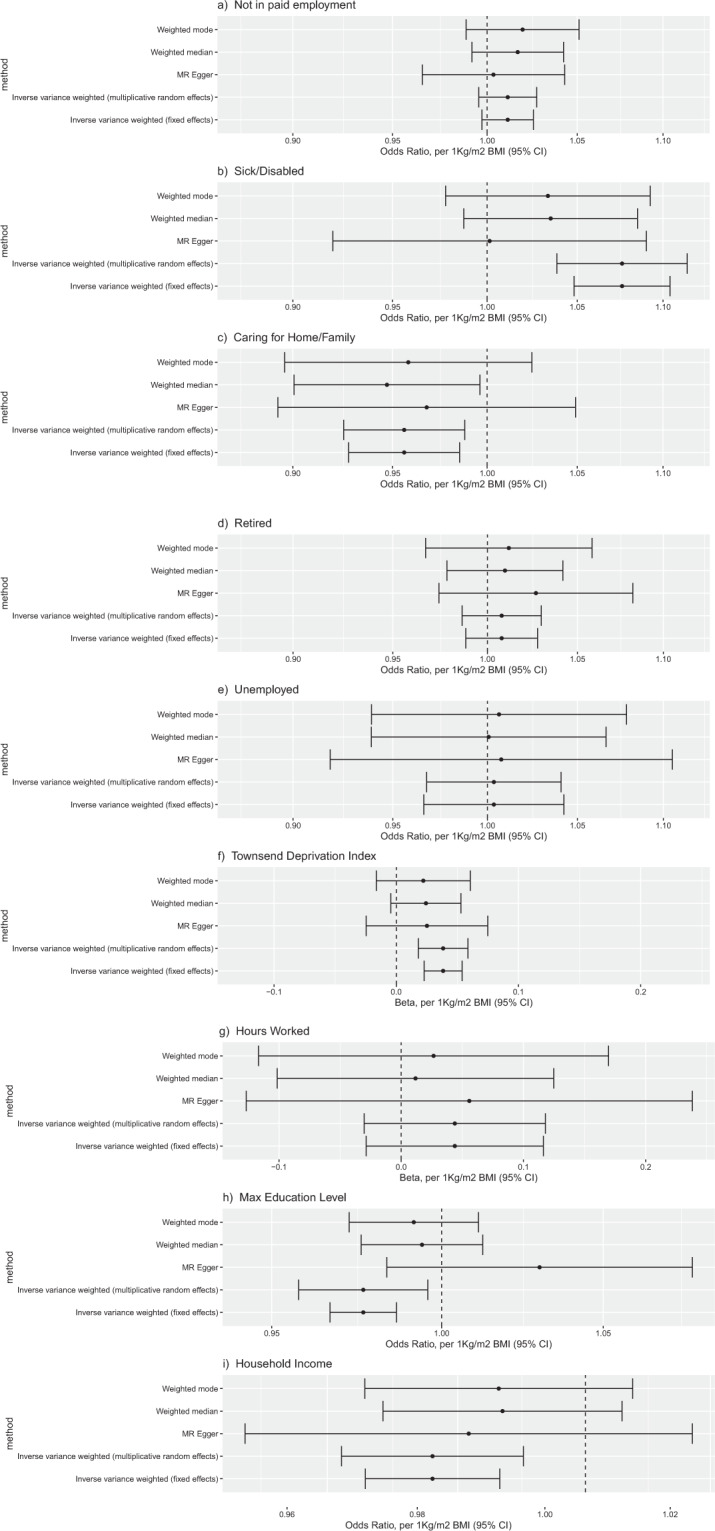
Forest plots of causal effect estimates of increased BMI on employment outcomes. Causal effect estimates for the effect of a one unit increase in BMI on: **a** not in paid employment, **b** sick/disabled, **c** caring for home/family, **d** retired, **e** unemployment, **f** Townsend Deprivation Index, **g** hours worked, **h** highest educational attainment, and **i** household income level.

Two-sample MR estimates of the causal effect of BMI on employment-related outcomes are presented in (1) Table 3 for the estimator selected via the Rücker model selection framework, and (2) Fig. 2 as forest plots for a subset of estimators. The estimates for all estimators are presented in Figs. S3, S4 and S5 and in Table S5, SS6 and SS7. For all outcomes except maximum education level, the set of estimates generated by the various estimators agreed with each other, in that there existed an interval lying within the 95% confidence intervals of all the estimates. For maximum education level, there are two sets of estimators which are internally consistent, but not consistent with each other. The split is determined by whether or not the estimator assumes balanced pleiotropy. In contrast to the observational associations (regressions), these MR analyses provide evidence for no (or very little) effect of BMI on early retirement, unemployment, non-employment, hours worked and educational attainment. These MR analyses also provide evidence that higher BMI (1) increased risk of sickness/disability (OR 1.076, 95% CI 1.039, 1.114), (2) decreased the odds of caring for home/family (OR 0.956, 95% CI 0.928, 0.985), (3) increased TDI (Beta 0.038, 95% CI 0.018, 0.059), (4) decreased household income level (OR 0.976, 95% CI 0.962, 0.990), these estimates reported being for the estimator selected via the Rücker model selection framework. For these outcomes, the MR causal effect estimates agree in sign with the observational associations. Full results are presented in Appendix 3.4. There was little evidence for causal effects of BMI on employment outcomes differing by sex, with *P* > 0.13 for all outcomes (Table 4). There was little evidence for causal effects of BMI on employment outcomes being moderated by age (full results are presented in Appendix 3.7).

**Table 3:** Two sample MR estimates of the causal effect of BMI on employment related outcomes.

Outcome	Method	Odds Ratio per 1Kgm2 increase in BMI	Odds Ratio 95% CI	Odds Ratio P Value	
Not in paid employment	Inverse variance weighted (fixed effects)	1.011	(0.9972, 1.025)	1.2E-01	
Sick/Disabled	Inverse variance weighted (multiplicative random effects)	1.076	(1.039, 1.114)	4.9E-05	*
Caring for Home/Family	Inverse variance weighted (fixed effects)	0.956	(0.9277, 0.9852)	3.4E-03	*
Retired	Inverse variance weighted (fixed effects)	1.008	(0.9883, 1.028)	4.4E-01	
Unemployed	Inverse variance weighted (fixed effects)	1.003	(0.9661, 1.042)	8.6E-01	
**Outcome**	**Method**	**Beta per 1Kgm2 increase in BMI**	**Beta 95% CI**	**Beta P Value**	
Townsend Deprivation Index	Inverse variance weighted (multiplicative random effects)	0.038	(0.01807, 0.05862)	2.1E-04	*
Hours Worked	Inverse variance weighted (fixed effects)	0.044	(−0.02867, 0.1164)	2.4E-01	
**Outcome**	**Method**	**Odds Ratio per 1Kgm2 increase in BMI**	**Odds Ratio 95% CI**	**Odds Ratio P Value**	
Highest Educational Attainment	MR Egger	1.030	(0.9836, 1.079)	2.1E-01	
Household Income	Inverse variance weighted (multiplicative random effects)	0.976	(0.9622, 0.9903)	9.9E-04	*

All outcomes effects were adjusted for age, sex, study assessment centre, and genetic principal components. Effect for Household Income level was additionally adjusted for NinHouseholdWindsorised12.

**Table 4:** Evidence for sex differences in causal effects of BMI on employment related outcomes.

Employment Category	MR Method	Rucker Model Male	Rucker Model Female	Beta 1kgm2 Male	Beta 1kgm2 95% CI Male	Beta 1kgm2 Female	Beta 1kgm2 95% CI Female	Z diff PValue
Not in paid employment	Inverse variance weighted (fixed effects)	Y	Y	0.0194	(0.000523, 0.0383)	0.0057	(−0.0157, 0.0271)	3.5E-01
Sick/Disabled	Inverse variance weighted (fixed effects)	Y		0.0646	(0.0296, 0.0996)	0.1055	(0.0653, 0.146)	1.3E-01
Sick/Disabled	Inverse variance weighted (multiplicative random effects)		Y	0.0646	(0.0266, 0.103)	0.1055	(0.0589, 0.152)	1.8E-01
Caring for Home/Family	Inverse variance weighted (fixed effects)	Y	Y	−0.0282	(−0.111, 0.0545)	−0.0489	(−0.0819, −0.016)	6.5E-01
Retired	Inverse variance weighted (fixed effects)	Y	Y	0.0073	(−0.016, 0.0305)	0.0109	(−0.0257, 0.0476)	8.7E-01
Unemployed	Inverse variance weighted (fixed effects)	Y	Y	−0.0047	(−0.0507, 0.0414)	0.0330	(−0.0348, 0.101)	3.7E-01
**Outcome**	**MR Method**	**Rucker Model Male**	**Rucker Model Female**	**Beta 1kgm2 Male**	**Beta 1kgm2 95% CI Male**	**Beta 1kgm2 Female**	**Beta 1kgm2 95% CI Female**	**Z diff PValue**
Townsend Deprivation Index	Inverse variance weighted (fixed effects)		Y	0.0331	(0.0116, 0.0546)	0.0422	(0.0193, 0.0651)	5.7E-01
Townsend Deprivation Index	Inverse variance weighted (multiplicative random effects)	Y		0.0331	(0.008, 0.0581)	0.0422	(0.0191, 0.0653)	6.0E-01
Hours Worked	Inverse variance weighted (fixed effects)	Y	Y	−0.0110	(−0.108, 0.0862)	0.0748	(−0.0346, 0.184)	2.5E-01
**Outcome**	**MR Method**	**Rucker Model Male**	**Rucker Model Female**	**Beta 1kgm2 Male**	**Beta 1kgm2 95% CI Male**	**Beta 1kgm2 Female**	**Beta 1kgm2 95% CI Female**	**Z diff PValue**
Highest Educational Attainment	Inverse variance weighted (fixed effects)	Y		−0.0254	(−0.0396, −0.0113)	−0.0256	(−0.0412, −0.00998)	9.9E-01
Household Income	Inverse variance weighted (fixed effects)	Y	Y	−0.0192	(−0.0337, −0.0046)	−0.0295	(−0.0457, −0.0133)	3.5E-01

Results are based on MR after exclusion of outlier SNPs. Results based on MR pre exclusion of outlier SNPs were similar.

The robustness of the causal effect estimates was investigated. Effect size heterogeneity across SNPs was suggested for being sick/disabled, TDI and maximum education level, even after removal of outlier SNPs from the instrument SNP set (Table S8). Consequently, fixed effects estimates and the maximum likelihood estimate may not be relied upon for these outcomes. Only for the maximum education level outcome was their evidence for unbalanced pleiotropy (Table S9). This suggests only the Egger estimators should be relied upon for this outcome. In other words, there may be unbalanced horizontal pleiotropy which only the Egger estimate accounts for. The Rücker model selection framework was in line with the results of the heterogeneity and unbalanced pleiotropy tests in its selection of fixed/random effect estimators and IVW/Egger estimators (Table S10). For each outcome, $I_{GX}^2$ was not lower than 0.91, so above the 0.9 threshold below which caution is advised [22]. Low $I_{GX}^2$ can result in an inflated false-positive rate for the unbalanced pleiotropy test, but unbalanced pleiotropy was not indicated for any outcome except maximum education level (Table S9). Single SNP MR analyses and Leave one SNP out MR analyses did not suggest any single SNP driving causal effect estimates for any outcome except maximum education level. Repetition of these analyses following exclusion of instrument SNPs identified as outliers produced very similar results. None of these robustness checks provided evidence to negate our main findings. Robustness analyses are reported more fully in Appendix 3.5. Results for a specimen outcome, (Sick/Disabled) illustrating the effect of outlier SNP removal are presented in Figs. S6, S7, S8, S9, S10, S11 and S12. Figure S13 shows exposure-SNP association scatter plots for maximum education level (the outcome for which the most outlier SNPs were dropped).

## Discussion

We investigated the causal effect of BMI on employment-related outcomes in the UK Biobank study by utilising a set of genetic variants robustly associated with increased BMI. Observational analyses (which are more subject to bias) indicated that among working aged people over 40, increased BMI was associated with a lower likelihood of being in work, with more working hours if people were in paid employment, living in a more deprived area, and with less income and education.

Estimates from MR analyses suggested that higher BMI increased the risk of not working due to sickness or disability, living in a more deprived area, lower household income and reduced the likelihood of caring for home/family. Estimates from MR analyses suggested little or no effect of BMI on early retirement, unemployment or hours worked (among the employed). The difference between the multivariable-adjusted regression-based approach to analysis and the MR analyses is noteworthy. In particular, the MR estimates systematically differed from the regression-based estimates which suggests that the multivariable-adjusted estimates may be affected by reverse causation or confounding.

Labour markets are well known to show strong gender patterns, potentially reflecting role differences, ubiquitous in many societies including the UK. We found prevalence differed across the sexes for several outcomes (especially the unemployed and caring for family/carer employment categories). However, there was little evidence for the causal effects of BMI on employment outcomes differing by gender. Our finding of increased BMI being potentially causally related to a reduced likelihood of being a carer was unexpected. This result largely reflects an effect for women as the prevalence for caring for home/family differed between the sexes, with females dominating (1% male, 8% female). This could potentially be explained by reduced capacity to perform the work of caring for home or family. Alternatively, higher BMI in women has been found to be causal on lower probability of cohabiting with a partner [24], which in turn may associate with increased caring for home or family.

Our study has several important strengths. First, we have explicitly sought to address causality by taking advantage of genetic variants which tend to be unconfounded and cannot be subject to reverse causation. Second, we have studied a very large population-based sample. Third, we have distinguished between reasons for not working in our analyses. Fourth, we have used several analytical approaches to test the robustness of our results to potential violations of the MR assumptions. However, there are some important limitations to note. These spring largely from (1) the measure of adiposity used (BMI), (2) the outcomes related dataset (UK Biobank) and (3) the assumptions of two-sample MR. Our study has used BMI as an indicator for obesity, with the limitations of this measure well established. Despite this, it remains a very widely used and clinically relevant indicator. BMI is a continuous variable and the effects we report may not reflect mechanisms pertaining to extremes of the BMI distribution. In fact, our observational association (regression) analyses provide some evidence that employment outcomes may differ for individuals with particularly high or low BMI. For each of the employment status outcomes, the actual risk in the extreme low and/or high BMI strata tended to be higher than predicted by the regression (see Appendix 3.2 and Fig. S14). Non-linearity in the relationship between BMI and employment outcomes has been previously reported [25]. We did not investigate this due to lack of power, and because any non-linearity estimates generated would likely be biased by the not representative nature of our study sample. If the relationship between dependent and independent variables is linear, then regression coefficient estimates will not in general be biased by ascertainment of the study sample on regressors. However, that no longer holds when the relationship is non-linear. For instance, the quadratic function could look linear if only one arm of the function was seen. Our study sample is not representative of the UK population [26, 27]. There is a well-known selection bias in the UK Biobank participants who have tended to self-select so as to be healthier, wealthier and better educated than the general population, consistent with a ‘healthy volunteer’ effect [28]. This could result in our findings not generalising well to the broader population. However, UK Biobank risk factor-trait associations have been found to generalise to the UK population when the risk factor retains much of its variation, as is the case for BMI [29]. The self-selection of participants may also have induced collider bias in our findings [26]. However, this has been previously investigated and there is evidence that genetic variants in general are not correlated with a broad range of 96 behavioural, socioeconomic, and physiological baseline factors and so would be unlikely to be subject to strong selection biases [30]. Informative missingness is another potential source of bias. Only one of our outcomes (household income) suffers high levels of missing observations (see Table 1). Those with missing household income are more likely to be female, have lower educational attainment and not be in paid employment (Table S11). Nevertheless, these differences are small and we do not envisage such ascertainment will qualitatively change our findings. As we restricted our study sample to White UK subjects aged over 40 years, our results may not generalise well to other UK ethnicities or to early working age people. Confounding by population structure could also potentially introduce bias. To minimise this, we restricted our population to the White UK ethnic group and adjusted for 40 GPCs plus assessment centre. Investigating latent structure within UK Biobank, Haworth et al. found a similar set of covariates accounted adequately for the relationship between BMI polygenic score and birth location (their Table 1) [31], so we feel this is unlikely. Lastly, our results are shaped by contemporary UK labour market forces and may therefore not apply to other societies with markedly different employment patterns. Such societies may include historical UK. As in many other high-income countries, sedentary jobs dominate, and these may be less susceptible to adverse employment consequences than more physically active jobs. Another potential source of bias is dynastic effects [32]. If parents with a genetic propensity for high BMI conferred some advantage/disadvantage regarding employment onto their offspring, this could result in association between BMI risk genes and offspring employment outcomes, i.e. confounding that could look like a casual effect for BMI in the current study.

Previous work investigating the relationship between weight and employment have produced conflicting findings. Methods to investigate the causal effect of weight on employment outcomes have included traditional cohort studies [33], twin studies [34], longitudinal studies [35, 36] and instrument variable studies. Instrument variables used have included area-based mean BMI [37], area-based obesity prevalence [38, 39], distance to closest exercise centre [39], child BMI [40, 41], mean family BMI [42], parental BMI [43], sibling BMI [44], lagged BMI [45] and genes (i.e. MR) [44, 46]. Results have been mixed with some studies finding evidence for BMI/obesity causing lower probability of paid employment and lower wages. This evidence tended to be stronger for obesity than for BMI. Inclusion of health covariates tended to have little effect on results suggesting discrimination as a possible route by which weight could affect employment outcomes [25, 42]. Kinge found BMI affected employment mainly through health. Sex differences have been reported [39, 42], and also it has been suggested that regional BMI level (against which an individual can be compared) plays a role [42].

Previous MR studies using UK Biobank have found higher BMI caused reduced socioeconomic position (higher deprivation, lower income, less years in education, lower odds of attaining a degree and lower odds of skilled employment), but did not find BMI causal on odds of employment [24, 47]. A randomised control trial used financial reward to create differences in weight loss in obese subjects. Weight loss was then used as an instrument on employment prospects [48]. Weight loss positively affected the employment prospects of obese women but not of obese men. Our results therefore add to the literature by providing evidence for a causal effect of BMI on employment, particularly increasing non-employment due to poor health. Our study complements previous UK Biobank MR studies by addressing effects in those of working age on different modes of not working.

Many studies have found unemployment and poor health to be associated, even after correction for socioeconomic position. The idea of health selection for unemployment has been tested by comparing the mortality rate in the unemployed against ‘wear-off’ rate by Clemens et al. [49]. They found health selection for unemployment was not supported. Similarly, in this study, there appears to be no effect of BMI on unemployment risk.

Our finding that BMI is causal on sickness/disability is plausible as BMI is a risk factor for many disorders. There are several mechanisms by which higher BMI might cause increased deprivation and reduced household income. A high BMI individual’s productivity could be reduced due to health issues. A high BMI individual may suffer workplace discrimination in hiring and promotion from work colleagues and employers, and stigma from customers if the position is public facing.

The adverse health consequences of obesity are well established. While there is a strong case to make for greater prevention efforts which will likely reduce future healthcare costs, as well as improving population health, policy responses to the growing epidemic of obesity have so far been inadequate [50]. Our study provides strong evidence that efforts to tackle high BMI will also help maintain a healthy workforce that is capable of contributing financially to society. In recent years there has been a rise in those of working age out of work due of sickness and disability in many high GDP countries, that is not matched by unemployment trends [51]. It is possible that excess weight might be contributing to this trend as obesity has risen concurrently with these labour market changes. Obesity and high BMI are also increasingly becoming socially patterned, with greater prevalence amongst socioeconomically disadvantaged groups. Our findings suggest that this is in part causal with higher BMI causing lower SEP and earnings. The adverse consequences for health inequity could be even greater than anticipated, with obesity exacerbating the social patterning of economic resources even further.

Our study provides evidence of higher BMI having detrimental causal effects putting people at risk of sickness/disability, reduced income and higher deprivation and this is a phenomenon supported for the broad range of BMI not just for the obese. This supports a role for government interventions aimed at shifting the BMI distribution of the whole population downwards. Interventions only targeted at obese individuals may also be useful, this study does not provide much evidence for or against such interventions. Further research is needed to better understand the shape of the relationship between BMI and social outcomes, including whether causal effects are qualitatively different in those with extreme BMI. Modelling studies which capture the broader societal benefits of tackling high BMI could demonstrate the potential for interventions to actually be cost-saving for societies and help inform future government policies.

We have found evidence of a causal relationship using the UK Biobank, however our estimates were based on a ‘white UK’ population, future research into the effects in other ethnic groups would be useful, as would research to compare effects across populations and societies with different labour markets. Furthermore, quantifying the population impact through the development of epidemiological and economic models could help inform debates regarding government’s role in reducing the obesity burden. Our finding that the adverse employment effects of higher BMI seem largely limited to those who experience disability, with no effects seen on unemployment, also raises the potential that early intervention through effective healthcare could mitigate the adverse societal impacts of obesity. Further research is required to explore whether this is the case, with collecting information on employment-related outcomes worth considering when evaluating new adiposity-related interventions or policies.

Supplementary information is available at the International Journal of Obesity website.

## Supplementary information

Appendix
