# Supplementary material for: Effects of increased body mass index on employment status: a Mendelian randomisation study
Source: Int J Obes (Lond). 2021 Jun 22;45(8):1790–801. doi: 10.1038/s41366-021-00846-x (PMC8310793; doi:10.1038/s41366-021-00846-x)
Supplement: Supplementary file 1 — Appendix [file 41366_2021_846_MOESM1_ESM.docx]

Appendix: Effects of increased body mass index on employment status: A Mendelian randomisation study

# Purpose

This is the appendix for the paper

- Effects of increased body mass index on employment status: A Mendelian randomisation study

It provides further details to the paper and describes supplementary files.

Table of Contents

[1 Purpose 1](#_Toc66290749)

[2 Methods 2](#_Toc66290750)

[2.1 Study population 2](#_Toc66290751)

[2.2 UK Biobank fields 2](#_Toc66290752)

[2.3 Genetic variants associated with obesity 3](#_Toc66290753)

[2.4 Polygenic score construction 4](#_Toc66290754)

[2.5 Regression of Outcomes on Exposure 4](#_Toc66290755)

[2.6 Regression of Exposure on Polygenic score 5](#_Toc66290756)

[2.7 SNP Associations with Outcomes 5](#_Toc66290757)

[2.8 MR Analyses 5](#_Toc66290758)

[2.9 Investigation of interaction with sex 6](#_Toc66290759)

[2.10 Investigation of interaction with age 6](#_Toc66290760)

[2.11 Outlier SNP removal 6](#_Toc66290761)

[3 Results 7](#_Toc66290762)

[3.1 Result Files 7](#_Toc66290763)

[3.2 Regression of Outcomes on Exposure 7](#_Toc66290764)

[3.3 Regression of Exposure on Polygenic score 8](#_Toc66290765)

[3.4 MR Analyses 8](#_Toc66290766)

[3.5 MR Robustness analyses 9](#_Toc66290767)

[3.6 Sex Stratified Analyses 11](#_Toc66290768)

[3.7 Age Band Stratified Analyses 11](#_Toc66290769)

[4 Discussion 11](#_Toc66290770)

[5 Figures 13](#_Toc66290771)

[6 Tables 28](#_Toc66290772)

[7 References 41](#_Toc66290773)

# Methods

## Study population

UK Biobank participants were excluded for the following reasons

- Not white UK
- Over retirement age at time of assessment (60 female, 65 male)
- Participant did not have a value for one of the employment outcomes of interest.
- mismatch between self-declared sex and sex genetically predicted
- abnormal number of X and Y chromosomes
- Low genotyping rate (<98.5%), which is indicative of low quality DNA
- Excess heterozygosity, which is indicative of DNA sample contamination
- Participant withdrawn
- Over-relatedness: if a pair surviving the above exclusions were too genetically related (kinship > 0.042, e.g. closer than 2^nd^ cousins), then one of the pair was dropped. The participant with the most relations was preferentially dropped. If both participants had the same number of relations then the subject dropped was randomly chosen.

A STROBE flowchart (Figure S1) reports the numbers lost through these exclusion criteria. The retained sample (230,791 participants) was the basis of all further analysis.

## UK Biobank fields

A list of the important UK Biobank fields used in this study is given in

Table S2.

## Genetic variants associated with obesity

We constructed a genetic instrument to investigate the effect of exposure BMI on occupation outcomes. Figure S2 shows a flowchart for this. This instrument was based on a set of Single Nucleotide Polymorphisms (SNPs) reported by Locke *et al.^1^* which were associated with BMI (P value < 5 x 10^-8^). The candidate set of associations was extracted via the gwas_catalog function of the R package MRInstruments from the MRBase catalogue of GWAS results^2^. These SNP associations (at P value < 1 x 10^-5^) were estimated in one or more of 4 cohorts, these being

- European and other ancestry cohort (up to 339,224 subjects)
- European ancestry cohort (up to 322,154 subjects)
- European ancestry female cohort
- European ancestry male cohort

Some SNPs had associations reported in more than one cohort. As one might expect, associations tended to be more precisely estimated in the larger dataset. For such SNPs, we retained the association reported in the largest dataset preferentially over the smaller dataset associations. After this, we were left with 183 unique SNP associations.

The association results downloaded from MRBase were incomplete in that for a few SNPs, one allele was not supplied (“other_allele” field was empty). For each such SNP, we found the missing allele reported in the Locke *at al.* SNP information. We also checked these alleles in the UKB genetic dataset (.bim file). This was done because otherwise the MR analysis software (TwoSampleMR R package^2^) would consider the SNP invalid and drop it. These SNPs were nearly all tri-allelic according to dbSNP. Presumably if they had survived the UK Biobank and Locke *et al*. genotype calling quality control then they were bi-allelic or very nearly bi-allelic in these datasets.

Associations not reaching genome-wide significance (p-value = 5 x 10^-8^) were then discarded. The following SNP exclusion criteria were then applied

- Hardy Weinberg Equilibrium (HWE): We used Family Wide Error Rate (FWER) = 1 Bonferroni correction to screen for SNPs not in HWE. In other words, we would expect one perfectly good SNP to be rejected according to this criterion.
- Low information content: Information score ranges from 0 to 1 and reflects the quality of sequencing. SNP dropped if < 0.9
- Low Minor Allele Frequency (MAF): SNP dropped if MAF < 0.01
- Palindromic and high MAF: Palindromic SNP dropped if MAF > 0.4

A SNP (rs1558902) in the FTO gene was retained despite marginally failing the palindromic SNP criterion above.

Linkage Disequilibrium (LD) clumping of the SNP set was used to identify a set of 77 mutually independent SNPs. This was done using using the ieugwasr R package ld_clump function with default clumping window size of 10 Mbases and a cut-off of R^2^ < 0.01. These SNPs (listed in Table S1) constituted our instrument SNP set for subsequent Mendelian Randomisation analyses.

## Polygenic score construction

In order to measure the strength of our genetic instruments and to validate the instrument-exposure association assumption of the Mendelian randomisation method, we generated a polygenic score for the exposure for each participant. This was constructed as the number of risk alleles the subject carried for a SNP, summed over all instrument SNPs. Scores were generated using PLINK’s score function^3^. We applied the default settings which impute any missing genotype with its expectation, (i.e. twice the SNP’s risk allele frequency).

We appreciate that the choice of using an unweighted score or a weighted score remains somewhat disputed. Our principal reason for using the unweighted score was simplicity, an analysis using weighted scores may have modestly higher power, but this is unlikely to have substantially affected the inferences we draw from our results. The polygenic score analysis aims to show association between the instrument set of SNPs and the exposure (i.e. that one of the MR assumptions is satisfied). A weighted score may be more efficient in this regard but this being demonstrated by the unweighted score does not detract from the assumption having been supported.

## Regression of Outcomes on Exposure

We regressed the outcomes on the exposure using the following regression equation.

$$Outcome=Exposure+sex+age+assessmentCentre+GPC1+GPC2+\ldots+GPC40$$

Where

- Exposure = BMI
- Sex – coded as male =1, female =2
- AssessmentCentre – the UKB assessment centre (UK Biobank data-field 54). These were represented in the regression by a set of dichotomous dummy variables.
- GPC1 … GPC40 – genetic principal components

The UK Biobank applied Principal Component Analysis to their subject genotype data and from this constructed 40 genetic principal components (GPCs). They have made per subject scores on these 40 GPCs available to researchers. These are in effect ancestry/ethnicity scores which characterise a person in the context of the genetic variation present in the sample population. Inclusion of GPCs as covariates in a regression of a trait on a genetic variant is a way of correcting for confounding between the genetic variant and the trait. In genetic epidemiology such confounding is also known as population stratification.

For ordinal, binary and continuous outcomes we used ordinal, logistic and linear regression respectively. The logistic and linear regressions were implemented using PLINK 1.9,^3^ the ordinal regressions were implemented using the polr function of the MASS R package. For some categories of the non-continuous outcomes, the count was low or even zero for some assessment centres. This would cause the regression to fail or return inaccurate estimates for these assessment centres. To overcome this, assessment centres were repeatedly merged until the minimum such count exceeded 50 prior to performing regressions. In all of the outcomes, at most three assessment centres were merged in this way.

We also investigated whether there is evidence for a non-linear relationship between BMI and each outcome. We did this by plotting predicted versus actual mean value for outcome per sex, in strata determined by dividing the BMI distribution into 20 quantiles.

## Regression of Exposure on Polygenic score

We regressed exposure on the polygenic score for the exposure, using linear regression. The regression equation was

$$Exposure=PolygenicScore+sex+age+assessmentCentre+GPC1+GPC2+\ldots+GPC40$$

BMI has a right skewed distribution, and as the regression only partially explains BMI, its residuals were also right skewed. Because of this, regression coefficients were estimated with robust standard errors. We also conducted ANOVA and calculated adjusted partial R^2^ for each regressor.

To investigate concerns about weak instrument bias we also calculated the F statistic for each instrument SNP. We did this by converting the SNP’s association p-value assuming an F (1, Infinity) distribution, this gives slightly lower values of F than an F (1, 2x10^5^) distribution would.

## SNP Associations with Outcomes

We regressed each outcome on each SNP of the instrument set using the UK Biobank dataset. The regression equation was

$$Outcome=riskAlleleCount+sex+age+assessmentCentre+GPC1+GPC2+\ldots+GPC40$$

Where

- $riskAlleleCount$ – the risk allele count (0, 1 or 2) for the SNP in question

We used ordinal, logistic and linear regression for ordinal, dichotomous and continuous outcomes respectively. The logistic and linear regressions were implemented using PLINK 1.9^3^, the ordinal regressions were implemented using the polr function of the MASS R package. For some categories of the non-continuous outcomes, the count was low or even zero for some assessment centres. This would cause the regression to fail or return inaccurate estimates for these assessment centres. To overcome this, assessment centres were repeatedly merged until the minimum such count exceeded 10 prior to performing regressions. In all of the outcomes, at most three assessment centres were merged in this way.

## MR Analyses

We conducted a two-sample MR analysis using the SNP-exposure associations reported by Locke *et al.^1^* and the SNP-outcome associations from the study sample. We estimated causal effects using the wide range of MR causal effect estimation methods made available by the TwoSampleMR R package.^2^ Heterogeneity amongst the causal effect estimates from the instrument SNPs was assessed with Cochran’s Q (assuming balanced pleiotropy) and Rücker’s Q (assuming unbalanced pleiotropy) using the RadialMR R package.^4^ Using Cochran’s Q and Rücker’s Q as inputs, we applied the Rücker model selection framework to identify the best fitting model between fixed effect and random effect versions of the IVW and Egger methods.^5^ We followed Bowden et al.^4^ in using 0.05 as a significance threshold for detecting pleiotropy for model selection purposes (see their Box 3). We conducted unbalanced pleiotropy tests (implemented via TwoSampleMR::mr_pleiotropy_test). We also calculated $I_{GX}^{2}$, a measure of the degree of violation of the No Measurement Error (NOME) assumption for SNP-exposure associations (implemented via TwoSampleMR::Isq).

## Investigation of interaction with sex

Given the well-established differences in employment by sex, we conducted sex-stratified MR analyses. We tested whether there was evidence for difference in causal effects between the sexes for each outcome. We did this using Fisher’s z-score method (a Wald test), comparing z to a standard normal distribution, by applying:

$$z=\frac{b_{Male}-b_{Female}}{\sqrt{{se}_{Male}^{2}+ {se}_{Female}^{2}}}$$

Where

- $b$ = causal effect estimate
  - for dichotomous outcomes = log odds ratio
  - for continuous outcomes = beta (regression coefficient)
- $se$ = standard error of $b$
- subscript denotes stratum

The Rücker model selection framework was used to identify which causal effect estimate to use per stratum outcome combination.

We used the same instrument SNP set for the sex stratified MR analyses as for the main MR analyses. An alternative would be to use sex specific instrument SNP sets based on the Lock et al. sex specific associations. We choose not to do this. The absence of sex-specific exposure associating SNPs from an instrument SNP set would weaken the instrument. MR based on such an instrument would be less precise but unbiased. The Locke et al exposure SNP association estimates are likely to be regressed towards the mean in the UK Biobank dataset. This could bias evidence for sex difference in causal effect if the degree of regression towards the mean differed between sexes. However, there is no reason to expect this given the Locke et al dataset and the UK Biobank dataset male to female ratios are similar.

## Investigation of interaction with age

We tested whether there was evidence for a moderating effect of age on the causal effect of BMI on each outcome. We stratified age into bands: 44 or less, 45-49, 50-55, 55-58, 59 or older. The lower bound of the oldest age band was set at 59, this allowed females as well as males to be in this stratum which simplified analysis. For each outcome for each MR method, we performed an F test to test whether the variance in regression coefficient estimates across age bands was large compared to the imprecision of the regression coefficient estimates.

## Outlier SNP removal

We used SNP contribution to the Cochran Q statistic to identify outlier SNPs, for each outcome. To visualise this we looked at quantile-quantile plots comparing the per SNP contributions to Cochran’s Q to a Chi^2^ df=1 distribution, Galbraith Radial plots, and radial funnel plots^4^. In Galbraith Radial plots, slope is interpretable as causal effect, vertical distance from point to regression line is interpretable as a SNPs contribution to Cochran’s Q, and horizontal value is interpretable as the SNP’s importance in the Inverse Variance Weighted (IVW) estimate (the square root of the SNP’s IVW weight). The latter two measure the influence of the SNP on the IVW causal effect estimate.

Outlier SNPs were detected and excluded from the instrument SNP set, then the MR analyses repeated using the reduced instrument SNP set. Egger Radial (implemented in egger_radial function of R package RadialMR^4^) was used to detect outlier SNPs. The outlier detection threshold was set at a false positive rate of 1 divided by the number of instrument SNPs. This should result in the Family Wise Error Rate (FWER) of 1. In other words, we should expect one perfectly good instrument SNP to be identified as an outlier for each outcome. We used this rather strict threshold as it resulted in multiple SNPs being classed as outliers and so gave different results from the Leave One SNP Out analyses.

# Results

## Result Files

A zip file is available on request that contains the full set of result files. Result files referred to in this document normally have names in the following form, or some subset thereof

- <<PREFIX>>_<<SUBJECTS>>_<<EXPOSURE>>_<<OUTCOME>>_<<OUTLIER>>*

Where

<<SUBJECTS>> indicates the subject dataset, and is one of

- ukb_17333_bmi_locke.subjects.qced – qc’ed UK Biobank subjects
- ukb_17333_bmi_locke.subjects.qced.Male – Male qc’ed UK Biobank subjects
- ukb_17333_bmi_locke.subjects.qced.Female – Male qc’ed UK Biobank subjects

<<EXPOSURE>> is

- f.21001.0.0 = BMI

<<OUTCOME>> indicates the employment outcome, and is one of

- iSickNotEmp - indicates the Sick/disabled outcome
- iFamilyNotEmp - indicates the Caring For Home/Family outcome
- iRetiredNotEmp - indicates the Early Retirement outcome
- iUnempNotEmp - indicates the Unemployed outcome
- iOtherNotEmp - indicates the Not in Paid Employment outcome
- f.767.0.0 - indicates the Work Hours Weekly outcome

<<OUTLIER>> indicates the instrument SNP set, and is one of

- incOutlier – results for the full SNP instrument set
- excOutlier – results for the reduced SNP instrument set (excluding outlier SNPs)

## Regression of Outcomes on Exposure

The strongest associations from the regressions of outcomes on BMI are presented in Table S3.

These results are also to be found in files

- collateRegressOutcomeOnExposure_ukb_17333_bmi_locke.subjects.qced_ageSexCentreGpc_OutcomeRegressor.csv
- collateRegressOutcomeOnExposureLinear_ukb_17333_bmi_locke.subjects.qced_ageSexCentreGpc_OutcomeRegressor.csv
- collateRegressOutcomeOnExposureOrdinal_ukb_17333_bmi_locke.subjects.qced_ageSexCentreGpc_Regressor.csv
- collateRegressOutcomeOnExposureOrdinal_ukb_17333_bmi_locke.subjects.qced_ageSexNinHouseCentreGpc_Regressor.csv

Evidence for a non-linear relationships between outcomes and continuous regressors (e.g. BMI, age) was investigated using scatter plots of mean predicted versus actual regressand value, in strata determined by dividing the regressor distribution into 20 quantiles.

For the dichotomous outcomes there is a general pattern that the non-employment outcome is more prevalent in the extremely low and high BMI strata than predicted by the regression. This is shown for sickness/disability in Figure S14.

These figures are available in files matching

- regressOutcomeOnExposure_ukb_17333_bmi_locke.subjects.qced_<<OUTCOME>>_f.21001.0.0_ageSexCentreGpc.ActualVersusFitted.pdf

Non-linear relationships were also investigated in the sex-stratified analyses. The same pattern is observed, strongly in the case of Family/Caring. These largely undermine any significance one might give to the MR causal result for Family/Caring.

The figures for the sex stratified analyses are found in files matching

- regressOutcomeOnExposure_ukb_17333_bmi_locke.subjects.qced_<<OUTCOME>>_f.21001.0.0_ageSexCentreGpc.ActualVersusFitted_stratifiedBySex_.pdf

## Regression of Exposure on Polygenic score

The strongest associations from the regressions of BMI on polygenic score are presented in Table S4.

These results are also found in file

- regressExposureOnPolygenicScore_ukb_17333_bmi_locke.subjects.qced_ageSexCentreGpc_f.21001.0.0.csv

## MR Analyses

The two sample MR causal effect estimates for all the employment category outcomes are presented, in Table S5, Table S6 and Table S7. They results are also presented graphically in the left hand side (LHS) forest plots in Figure S3, Figure S4 and Figure S5.

For all outcomes (except Maximum Education Level), the causal effect estimates generated by the various methods agree with each other in that there exists an interval that lies within all the 95% confidence intervals. For Maximum Education Level, there are two sets of methods which give consistent estimates, but these two sets are not consistent with each other. The split is determined by whether the method assumes balanced pleiotropy or not.

The tables are also available in files

- collateMrAnalyses_ukb_17333_bmi_locke.subjects.qced_f.21001.0.0_<<A>>_<<B>>_AllCausalEffectEstimates_incOutlier.csv
- collateMrAnalysesBeta_ukb_17333_bmi_locke.subjects.qced_f.21001.0.0_<<A>>_<<B>>_AllCausalEffectEstimates_incOutlier.csv

The forest plots are available in files

- collateMrAnalyses_ukb_17333_bmi_locke.subjects.qced_f.21001.0.0_<<A>>_<<B>>_forestPlot_allMethods. pdf
- collateMrAnalysesBeta_ukb_17333_bmi_locke.subjects.qced_f.21001.0.0_<<A>>_<<B>>_ forestPlot_allMethods.pdf

## MR Robustness analyses

Robustness of the MR analyses results was investigated in several ways. Outlier SNPs were detected and excluded from the instrument SNP set, then the MR analyses repeated using the reduced instrument SNP set (see section 2.11). In addition, various tests were performed.

MR estimates based on instrument sets with outlier SNPs excluded, are presented graphically as forest plots in the right hand side of Figure S3, Figure S4 and Figure S5.

Heterogeneity test results are presented in Table S8.

Unbalanced pleiotropy test results are presented in Table S9.

Rücker model selection framework results are presented in Table S10.

These results are also available in files

- collateMrAnalyses_ukb_17333_bmi_locke.subjects.qced_f.21001.0.0_<<A>>_<<B>>_<<C>>_<<D>>. csv
- collateMrAnalysesBeta_ukb_17333_bmi_locke.subjects.qced_f.21001.0.0_<<A>>_<<B>>_<<C>>_<<D>>. csv

where

- <<A>> (outcome set identifier) = NotEmp | Ordinal | Beta
- <<B>> (covariates id) = ageSexCentreGpc | ageSexNinHouseCentreGpc
- <<C>> (result type) = HeterogeneityTest | BalancedPleiotropyTest | RuckerModelSelection | I2gx | SteigerTest | AllCausalEffectEstimates | RepresentativeCausalEffectEstimate
- <<D>> (SNP set) = incOutlier | excOutlier

In addition, for each outcome, the following diagnostic plots were generated

- scatterplot of SNP-outcome versus SNP-exposure association
- forest plot of causal effect estimates
- QQ plot of Single SNP causal effect estimates
- QQ plot of Leave One SNP Out causal effect estimates
- Rucker Model Selection Framework plot
- QQ plot of SNP Cochran Q
- Galbraith radial plot
- Galbraith radial funnel plot

These plots are available in files

- do2SampleMrAnalyses_ukb_17333_bmi_locke.subjects.qced_f.21001.0.0_<<Outcome>>_<<Covariates>>_<< snpSetId >>.pdf

where

- <<Outcome>> (outcome) = iSickNotEmp | iFamilyNotEmp | iRetiredNotEmp | iUnempNotEmp | iOtherNotEmp | f.767.0.0 (=Hours Worked) | f.189.0.0 (=TDI) | maxEducLevel | householdIncome
- << Covariates >> (covariates id) = ageSexCentreGpc | ageSexNinHouseCentreGpc
- << snpSetId>> (SNP set) = incOutlier | excOutlier

For all outcomes, the leave one SNP out analysis and per SNP analysis gave approximately Gaussian causal effect estimates, barring a few outliers. For most of the outcomes, the SNP’s contribution of Cochran’s Q followed a Chi^2^ df=1 distribution, barring a few outliers.

The most SNPs detected as outliers and removed was 11, 6 and 5 for Max Education Level, Household Income Level and the Sick/Disabled outcome respectively. For some outcomes (Sick/Disabled, Max Education Level, Household Income Level), the SNP’s contribution of Cochran’s Q appear to follow an inflated Chi^2^ distribution. For these outcomes, the outlier detection method was probably inappropriate, calling more SNPs outliers than warranted. In addition, for Max Education Level, the MR Egger Radial regression appears to misfit the data in that its regression line goes through the top of the scatterplot of exposure-SNP associations rather than the middle (see Figure S13. It seems the plot function draws the fit through the origin.

The MR causal effect estimates generated using the reduced instrument SNP set were very similar to those generated using the full set (compare left hand side versus right hand side plots in Figure S3, Figure S4 and Figure S5. Removal of outlier SNPs had little effect on the conclusions one would draw.

To illustrate the effect of outlier SNP removal we present the results for a specimen outcome, the Sick/Disabled outcome. Scatter plots of Sick/Disabled-SNP associations (from Locke et al.) versus exposure-SNP associations (estimated from our UK Biobank analytical sample) are presented in Figure S6. Quantile-Quantile plots comparing Single SNP and Leave One SNP Out causal effect estimates for BMI on the Sick/Disabled outcome against Gaussian distributions, are presented in Figure S7 and Figure S8 respectively. The SNP contributions to Cochran’s Q appear to follow an inflated Chi^2^ df=1 distribution (Figure S9). The removal of outlier SNPs did not change the Rücker model selection framework’s preferred model (IVW Multiplicative Random Effects) (Figure S10). The Galbraith radial plots and funnel plots are shown in Figure S11 and Figure S12 respectively. These plots are taken from file

- do2SampleMrAnalyses_ukb_17333_bmi_locke.subjects.qced_f.21001.0.0_iFamilyNotEmp_ageSexCentreGpc.pdf

A similar file is available for each outcome.

## Sex Stratified Analyses

MR analyses were repeated in male only and female only subsets of the study sample for all outcomes. The results for the sex stratified MR analyses were similar to those for the main MR analyses. A Wald test was used to compare the male only and female only causal effect estimates of BMI on each outcome. There was little evidence for differences in causal effect across sex (Table 4).

The full results for the sex stratified MR analyses are presented in files matching

- *_ukb_17333_bmi_locke.subjects.qced.Male_*
- *_ukb_17333_bmi_locke.subjects.qced.Female_*

The Wald test results are available in files

- collateORCausalEffectDiffBetweenStrata_MaleFemale_<<covariates>>_<<snpSet>>*csv
- collateBetaCausalEffectDiffBetweenStrata_MaleFemale_<<covariates>>_<<snpSet>>*csv

## Age Band Stratified Analyses

MR analyses were repeated in the age band defined strata. The results for the age band stratified MR analyses were similar to those for the main MR analyses. An F test was used to see whether age moderate causal effect of BMI on each outcome. There was little evidence for a moderating effect of age, with p > 0.04 for all combinations of outcome and MR method.

The F test results are presented in file

- testBetaCausalEffectDiffBetweenAgeBands_R.csv

# Discussion

Here we elaborate on limitations of our study.

Two Sample MR assumes the exposure-SNP and outcome-SNP associations are obtained from independent datasets. If these datasets overlapped, causal effect estimates could be biased. However, there is no subject overlap between the Locke *et al.* and UK Biobank datasets, so this assumption was met.

Ideally in MR analyses, the outcome-SNP and exposure-SNP regressions control for the same covariates. Locke *et al*. report that for each contributing dataset the covariates they adjusted for included age squared (Locke *et al.*, pg. 1;11). We omitted adjusting for age squared. When included as a covariate in our outcome-SNP association studies, the regressions (implemented in PLINK) failed. It transpired that age and age squared were highly correlated (0.98) and this caused the failure. In the regression of exposure on polygenic score (implemented in R), age squared when included as a covariate was a significant regressor, but made little difference to estimates. In light of this, we doubt the omission of age squared as a regressor had much impact on the MR results. The surprisingly high correlation can be shown by drawing a sample X from the age range 40 to 65, then correlating X with X squared.

The exposure-SNP association inputs are likely to be inflated by winner’s curse as we took our exposure-SNP associations from a discovery association study (Locke *et al*. report 56 of their 97 genome-wide significant loci as novel). This inflation would tend to deflate causal effect estimates and their significance. The estimate of $I_{GX}^{2}$ at around 0.91 implies a degree of regression dilution which again would tend to deflate the magnitude of our casual effect estimates.

For some outcomes (e.g., sickness/disability), individual SNP contributions to Cochran’s Q appear distributed as an inflated ChiSq df=1 distribution. This heterogeneity in casual effect estimates across SNPs indicates horizontal pleiotropy and/or un-modelled confounders of the SNP-outcome regression. Some of the estimation methods employed are robust to such heterogeneity, however these estimation methods make the Instrument Strength Independent of Direct Effect (InSIDE) assumption.

Selection bias is a concern for MR study designs. Such selection can induce paths between the casual ancestors of any variable influencing likelihood of study inclusion. If both exposure and outcome were such casual ancestors, then paths would be induced between instrument SNPs and outcome. This would bias the regression of outcome on SNP (via collider bias). Furthermore, the strength of such induced paths, and thence the regression bias, would depend on the SNP exposure association strength, violating the InSIDE assumption.

Is it likely that both exposure and outcome could be causal ancestors of likelihood for subject study inclusion? There is evidence of UK Biobank subjects being healthier than the general population, so the casual effect of the sickness/disability outcome on likelihood of study inclusion is probably negative. Let us postulate that BMI also has a negative causal effect on study inclusion. Collider bias through the selection on study inclusion would induce paths between outcome and SNPs in an instrument strength dependent way, violating the InSIDE assumption and biasing casual effect estimates. However, in this case the casual effect of both exposure and outcome on likelihood of study inclusion have the same (negative) sign. Because these signs are the same, the induced paths between outcome and SNPs (which have been arranged to have positive effects on exposure) would be negative. This would tend to attenuate the strength of regressions of outcome on SNPs in an instrument strength dependent way. Thus, although the InSIDE assumption would be violated, and methods assuming it would be biased, the bias would tend to be attenuation bias. Yet for sickness/disability we have causal effect estimates significantly different from zero. So, given the postulated effect of BMI on study inclusion, the suspected bias does not provide alternative explanations for the results seen.

# Figures

Figure S1 STROBE flowchart of UK Biobank participant exclusions


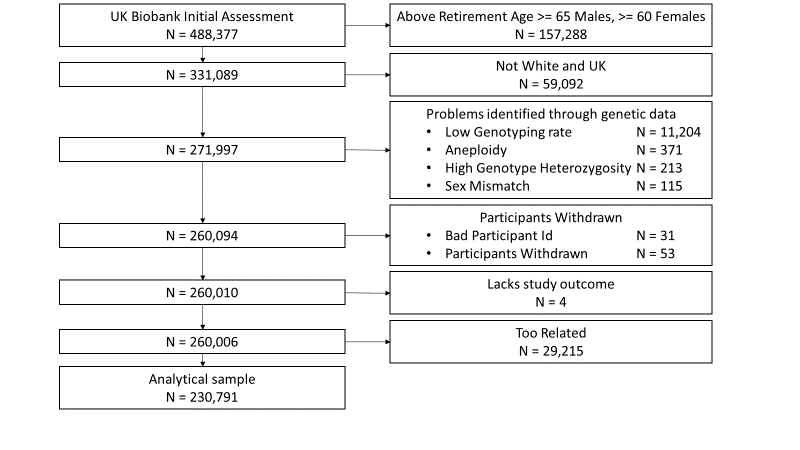


Figure S2 Flowchart of SNP screening for MR instrument


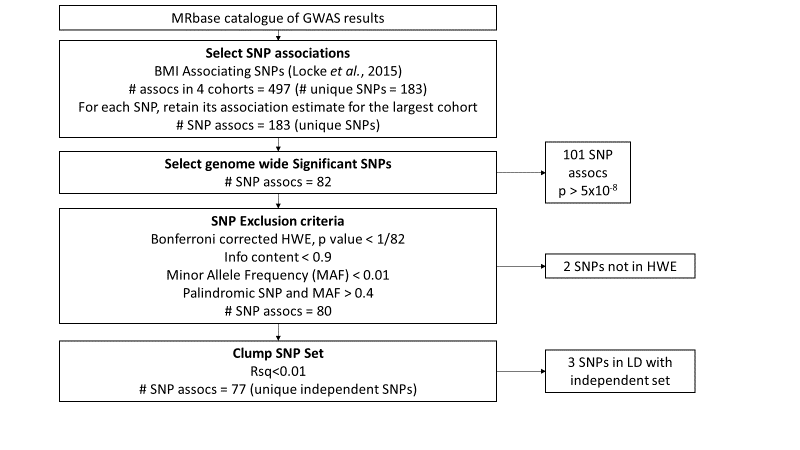


Figure S3 Forest plots of causal effect estimates for employment category outcomes.


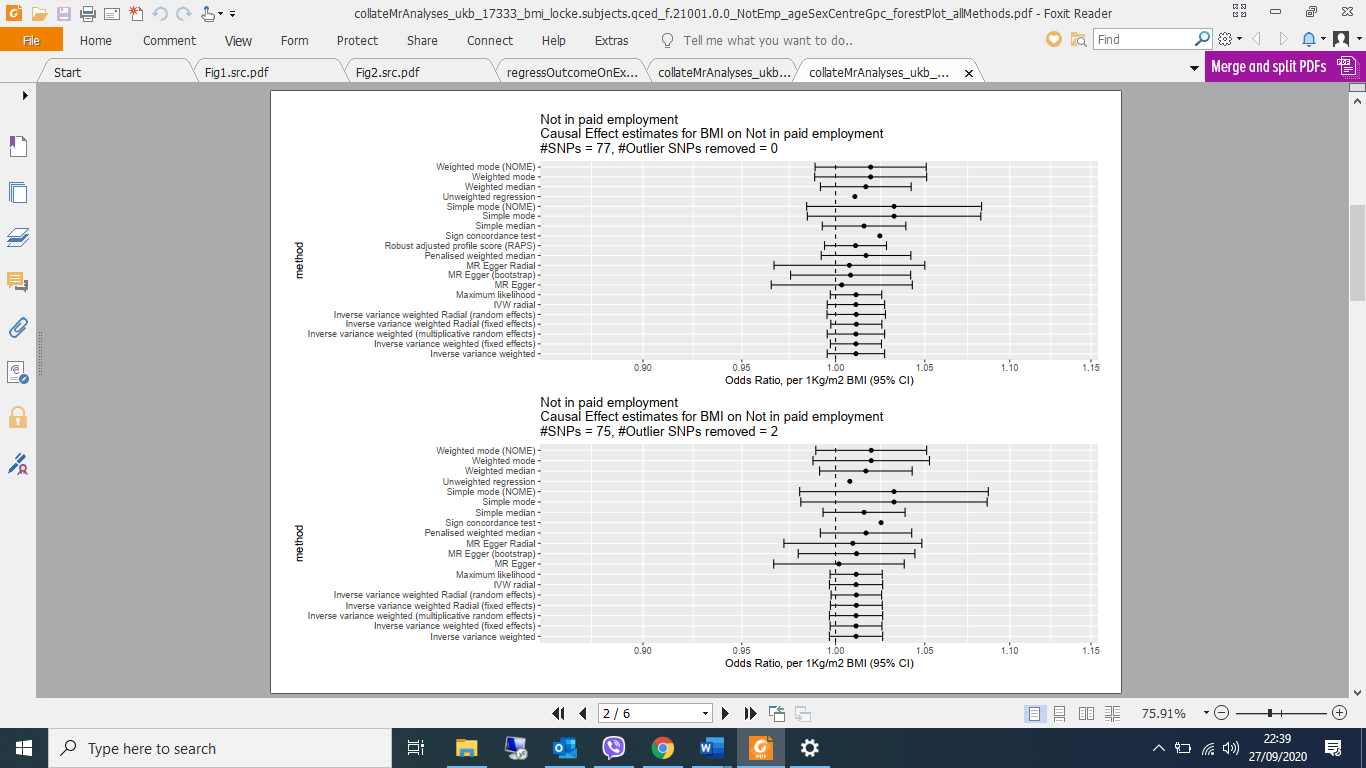


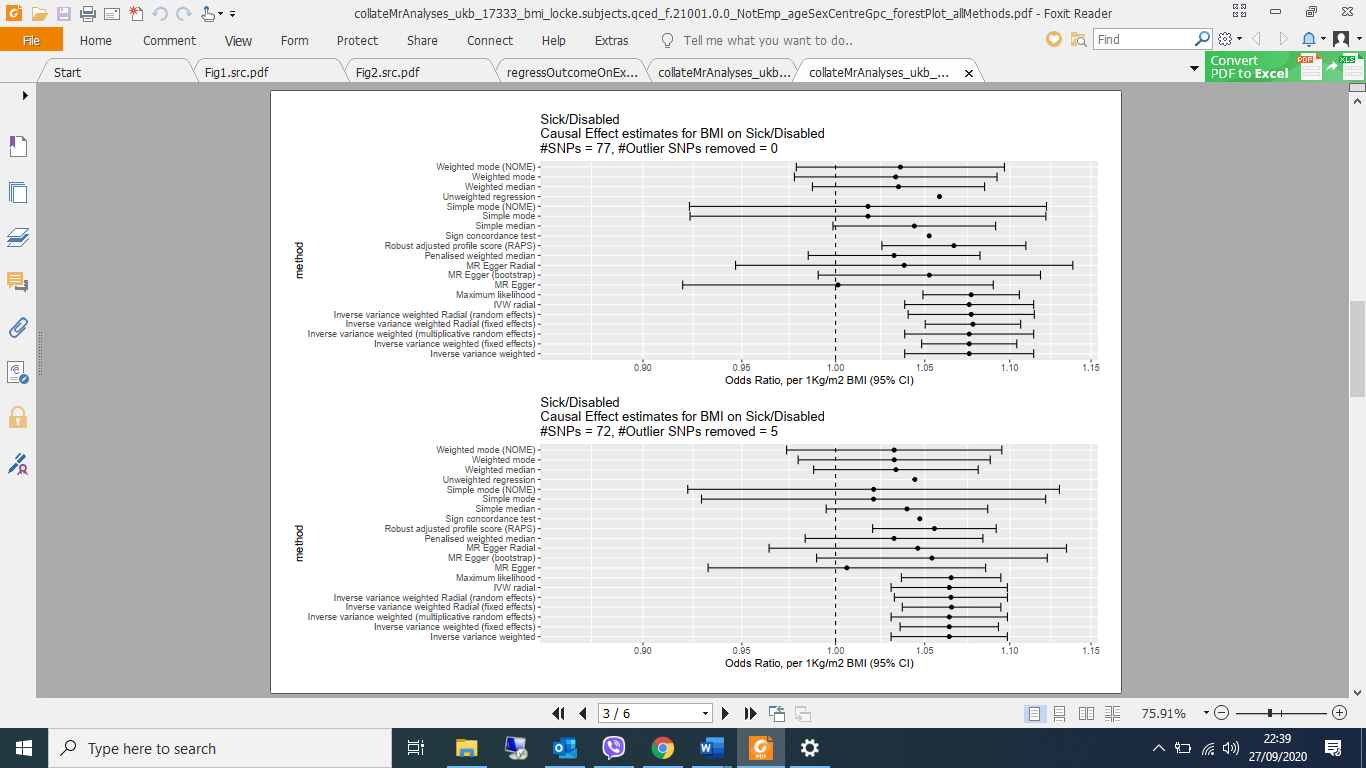


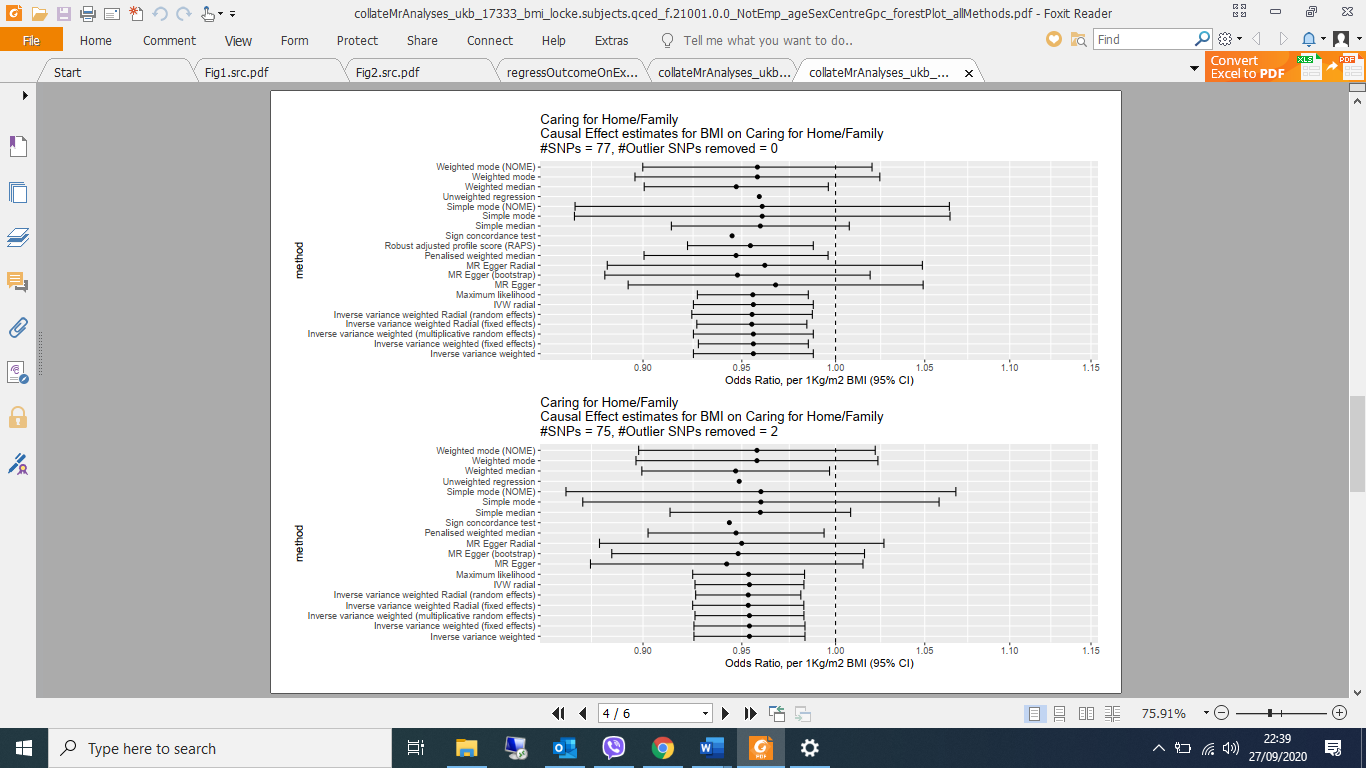


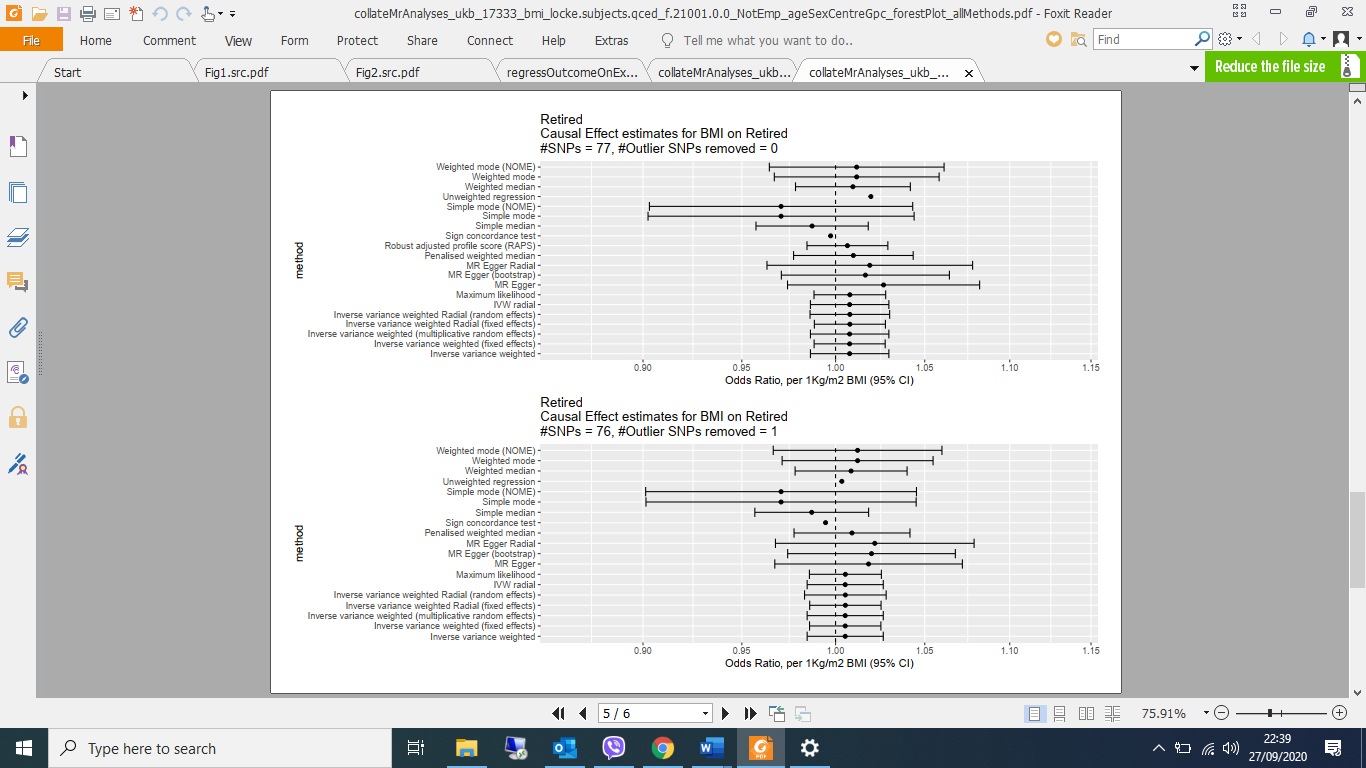


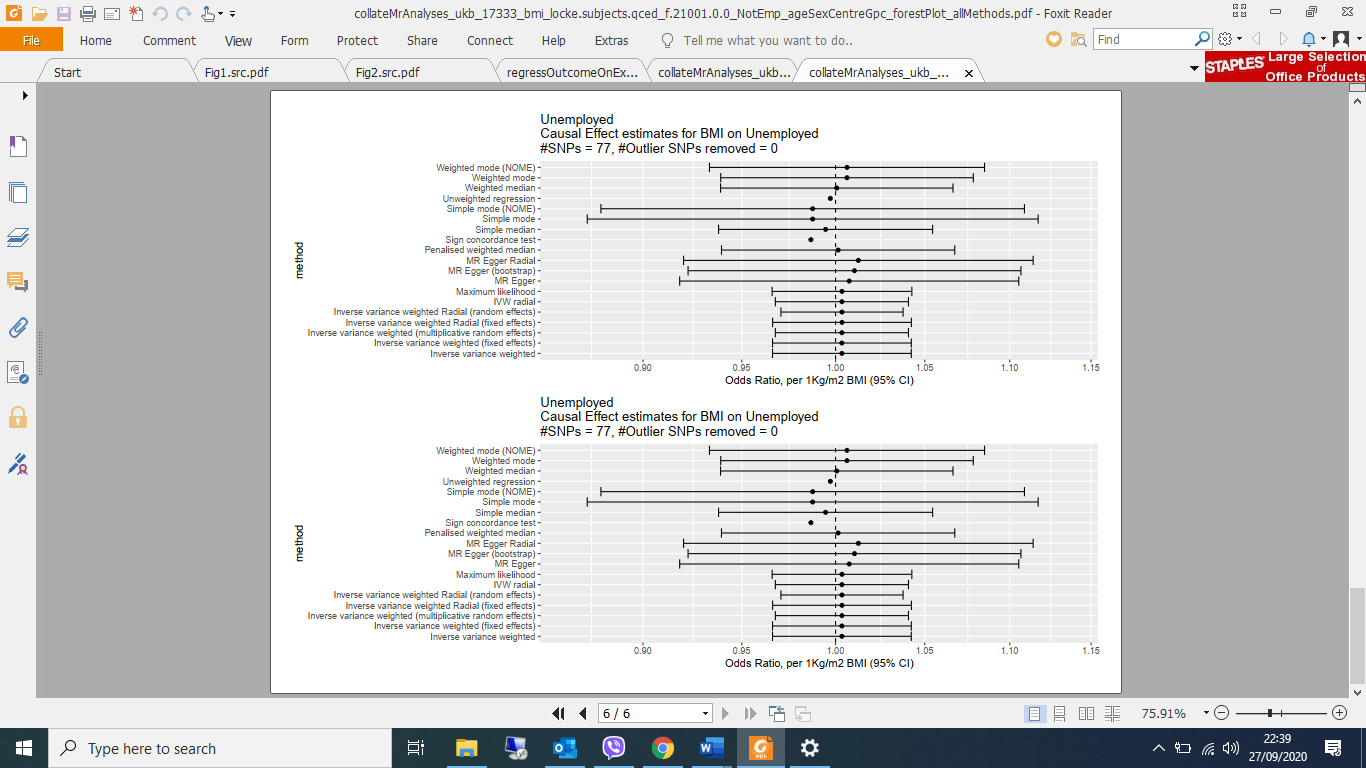


Footnote: Forest plots of causal effect estimates for employment category outcomes (full instrument SNP set). LHS – MR analyses results for full instrument set. RHS – MR analyses results for reduced instrument set. Causal effect estimate (plus 95% Confidence Interval) is presented per 1 Kg/m2 increase in BMI. Estimates for two of the methods appear without confidence intervals. The ‘sign concordance test’ method used did not return any precision estimate. The ‘Unweighted regression’ method estimates come with huge confidence intervals (not shown) rendering it pretty useless.

Figure S4 Forest plots of causal effect estimates for BMI on Weekly Hours Worked and TDI outcomes


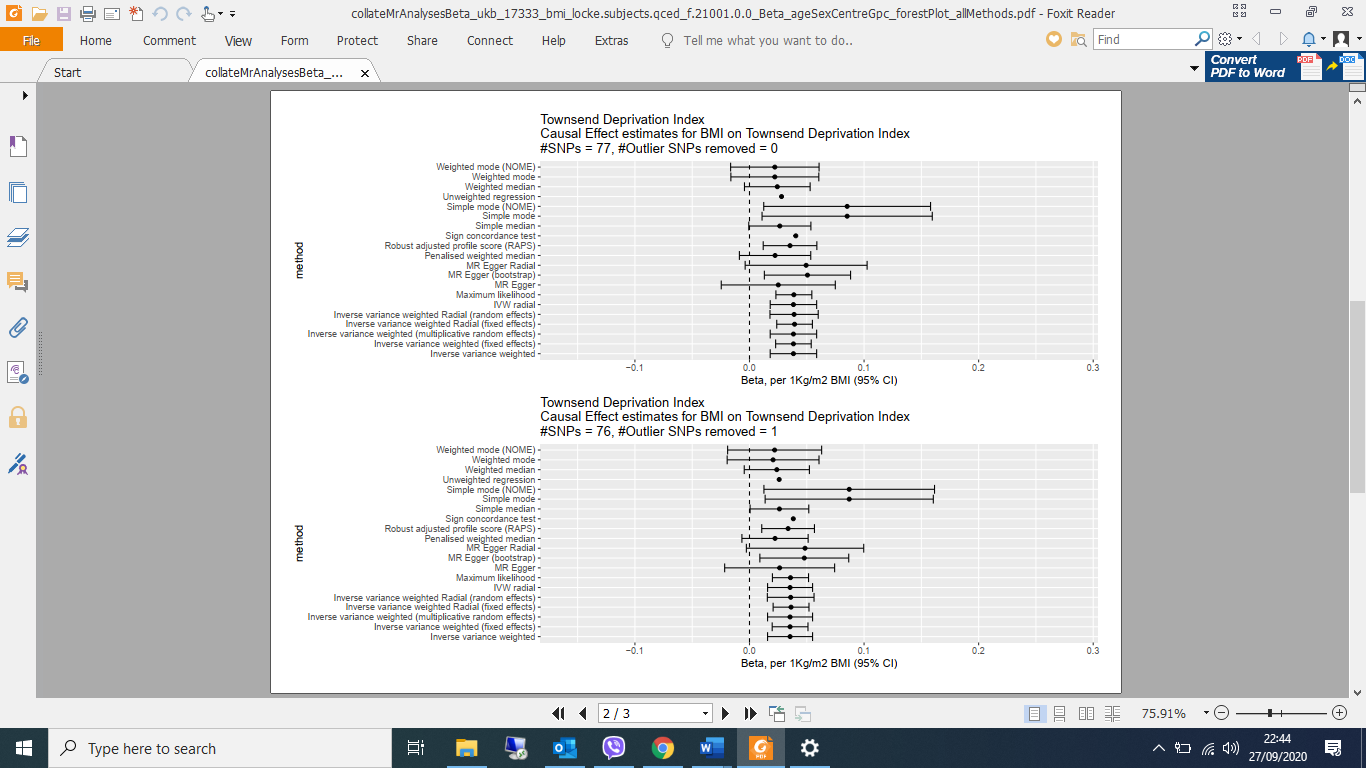


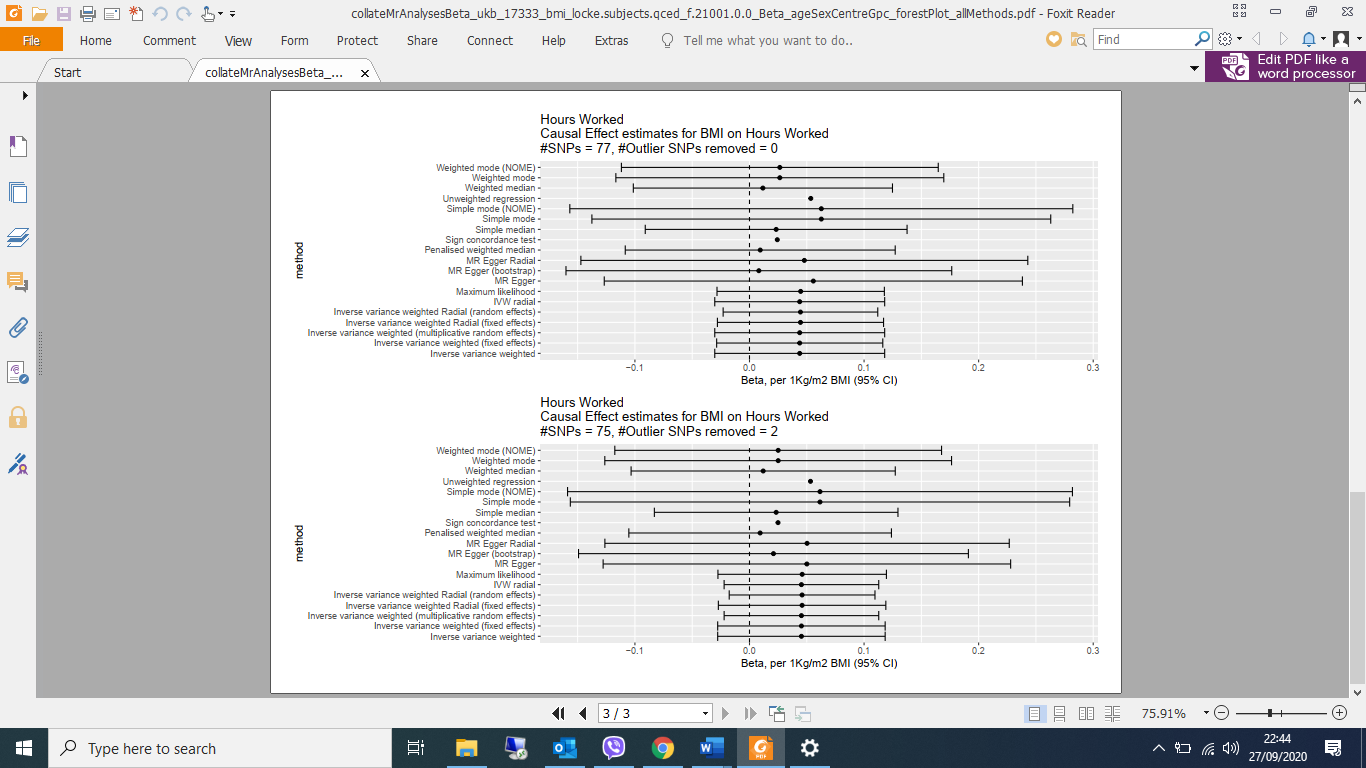


Figure S5 Forest plots of causal effect estimates for BMI on Household Income Level and Max Education Level


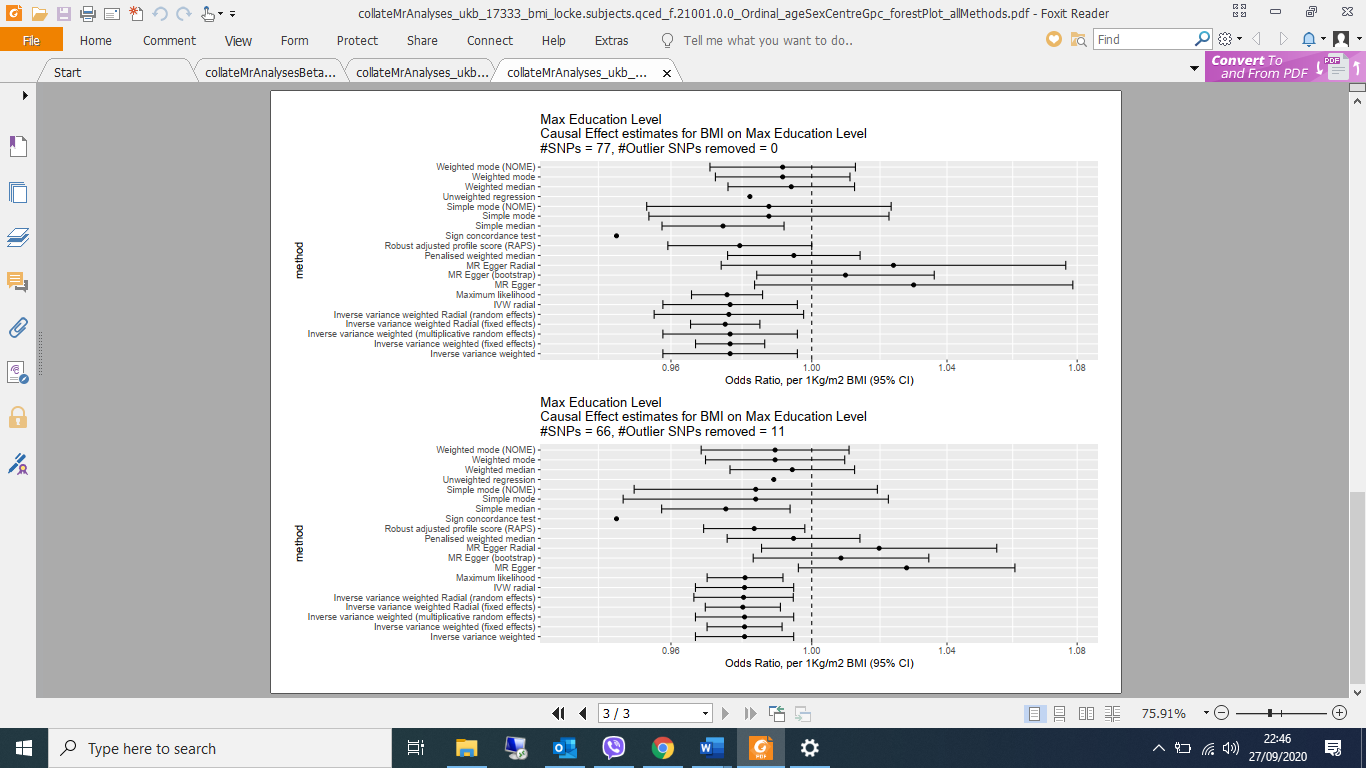


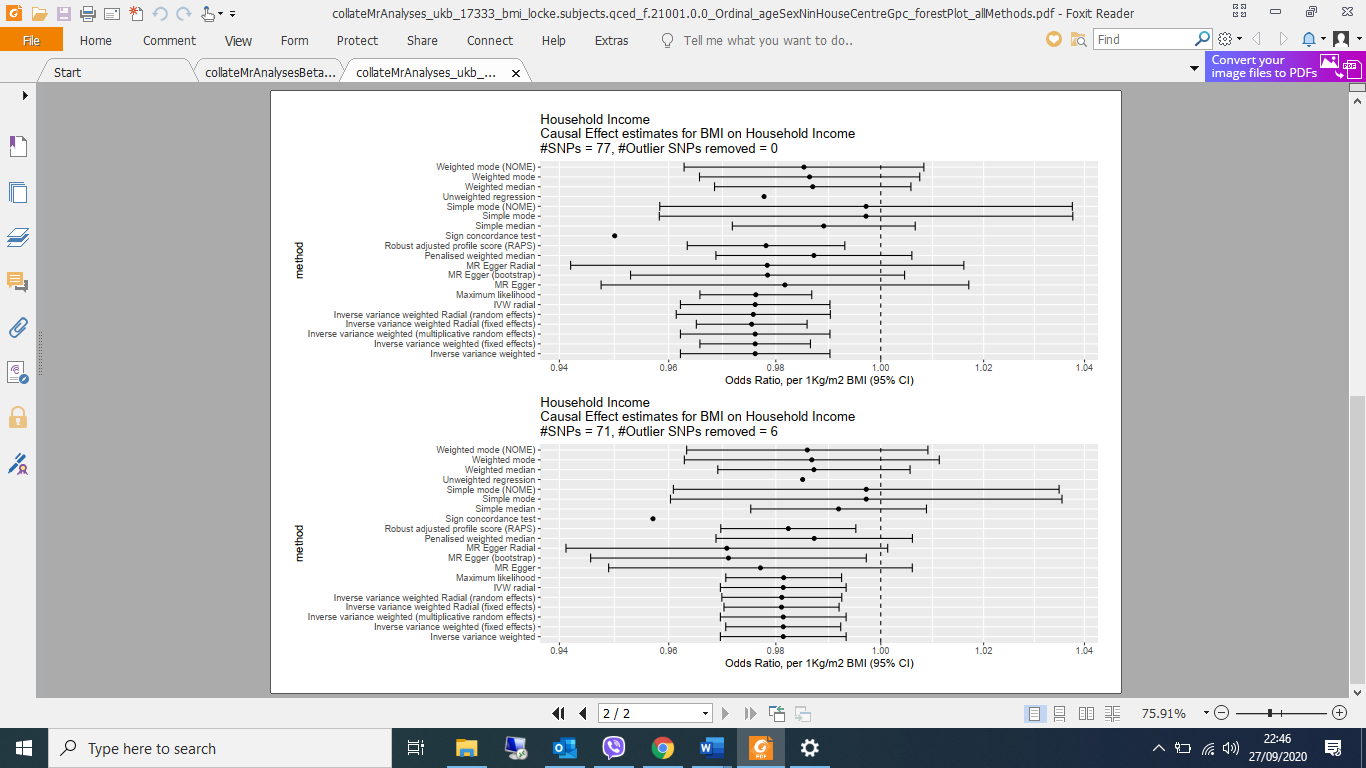


Footnote: Forest plots of causal effect estimates for continuous employment outcomes (full instrument SNP set). LHS – MR analyses results for full instrument set. RHS – MR analyses results for reduced instrument set. Causal effect estimate (plus 95% Confidence Interval) is presented per 1 Kg/m2 increase in BMI. Estimates for two of the methods appear without confidence intervals. The ‘sign concordance test’ method used did not return any precision estimate. The ‘Unweighted regression’ method estimates come with huge confidence intervals (not shown) rendering it pretty useless.

Figure S6 Scatter plots of Sick/Disabled-SNP associations versus exposure-SNP associations. LHS – complete instrument SNP set. RHS after removal of outlier SNP.


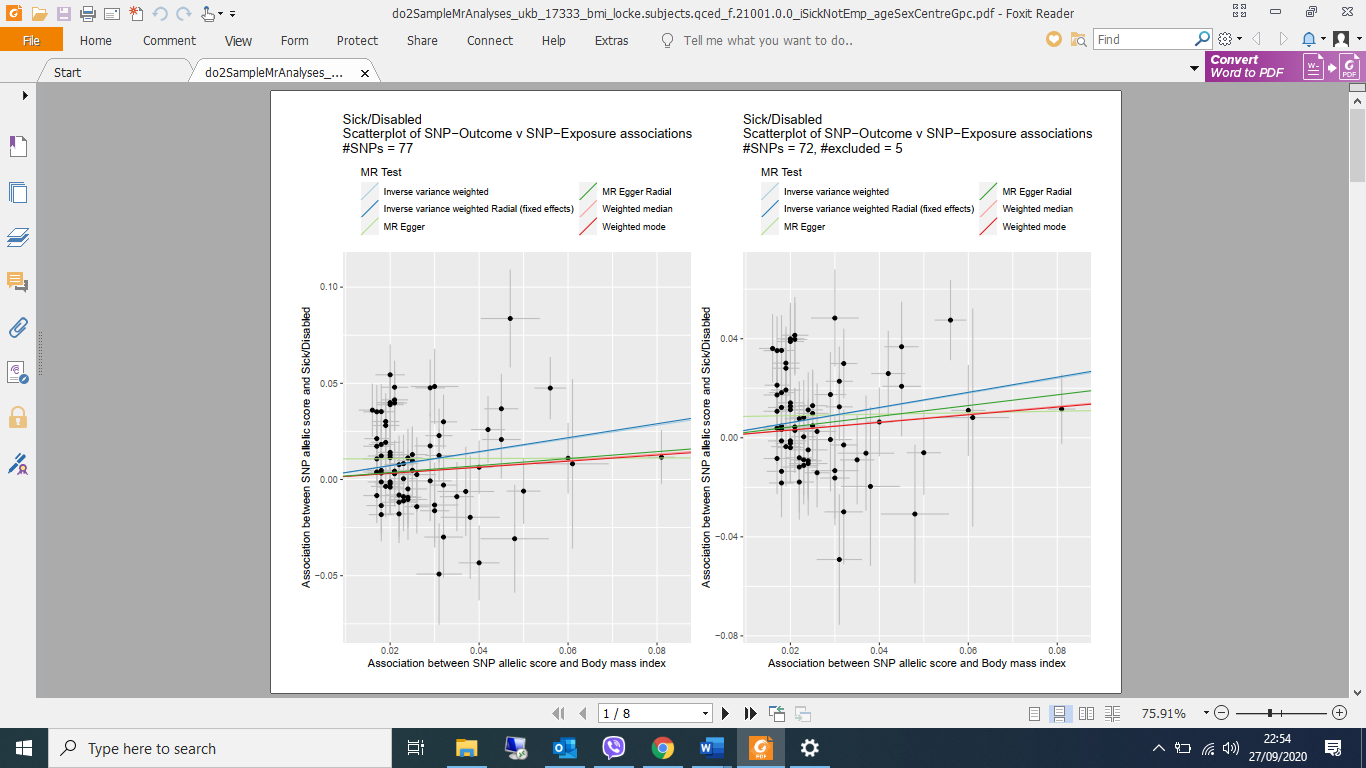


Scatter plot with x axis – BMI-SNP regression coefficient estimates from Locke et al. (normalised BMI), y axis – Sick/Disabled-SNP log odds from UK Biobank regressions. Also plotted are the fits for six causal effect estimation methods.

Figure S7 Quantile-Quantile plots comparing Single SNP causal effect estimates for BMI on the Sick/Disabled outcome against Gaussian distributions. LHS – complete instrument SNP set. RHS after removal of outlier SNP.


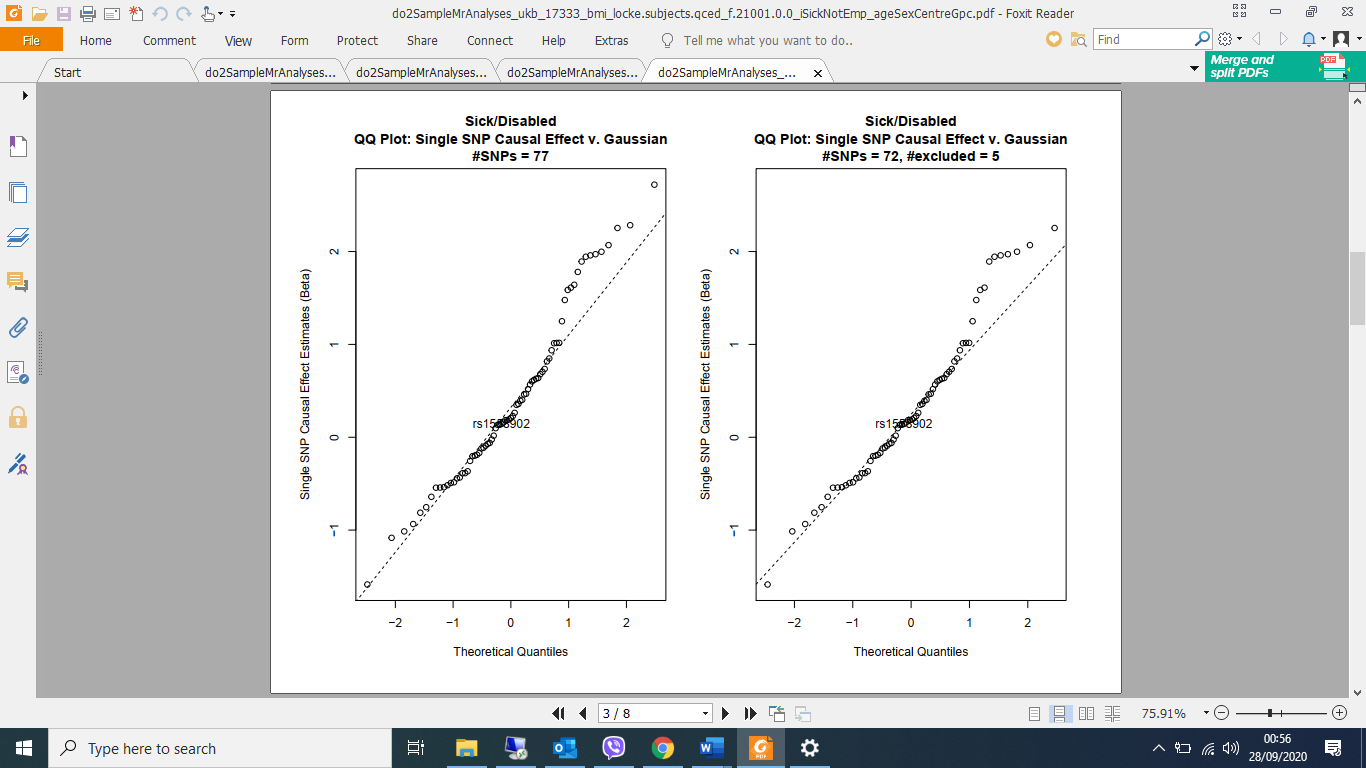


Footnote: Position of SNP rs1558902 from the FTO gene is marked.

Figure S8 Quantile-Quantile plots comparing Leave One SNP Out causal effect estimates for BMI on the Sick/Disabled outcome against Gaussian distributions. LHS – complete instrument SNP set. RHS after removal of outlier SNP.


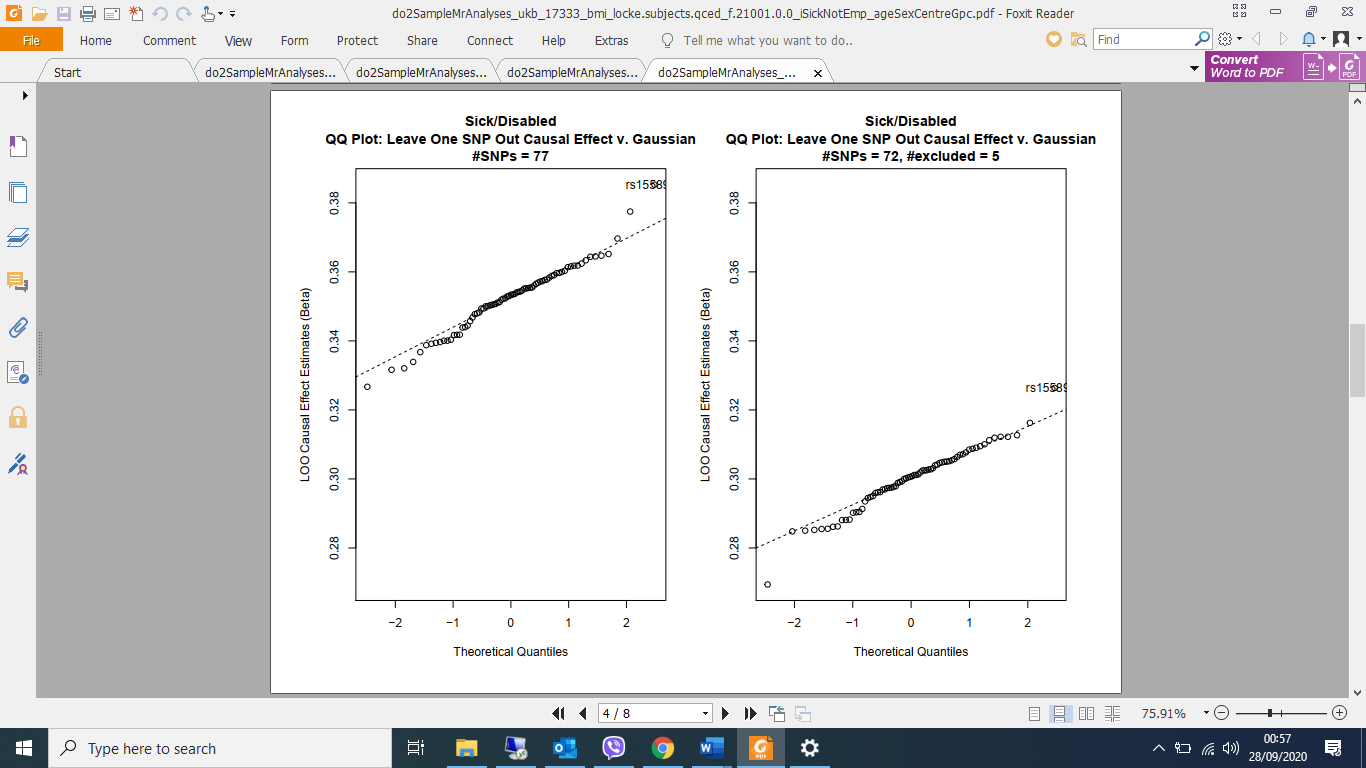


Footnote: Position of SNP rs1558902 from the FTO gene is marked.

Figure S9 QQ Plots comparing SNP contribution to Cochran’s Q to a Chi^2^ df=1 distribution for Sick/Disabled outcome. LHS – complete instrument SNP set. RHS after removal of outlier SNP.


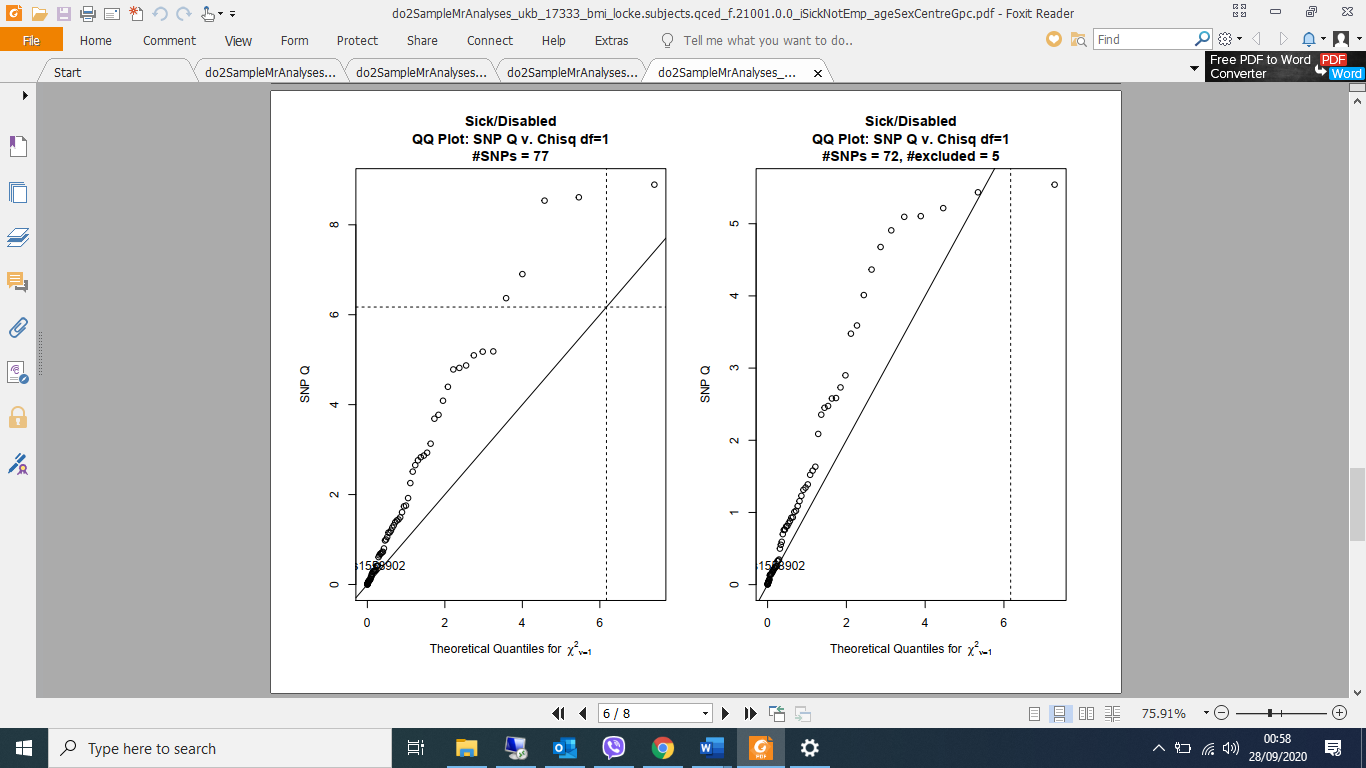


Footnote: The dashed horizontal line marks the threshold for outlier SNP detection. Position of SNP rs1558902 from the FTO gene is marked.

Figure S10 Rücker model Selection Framework Plots for Sick/Disabled outcome. LHS - complete instrument SNP set. RHS - after removal of outlier SNP.


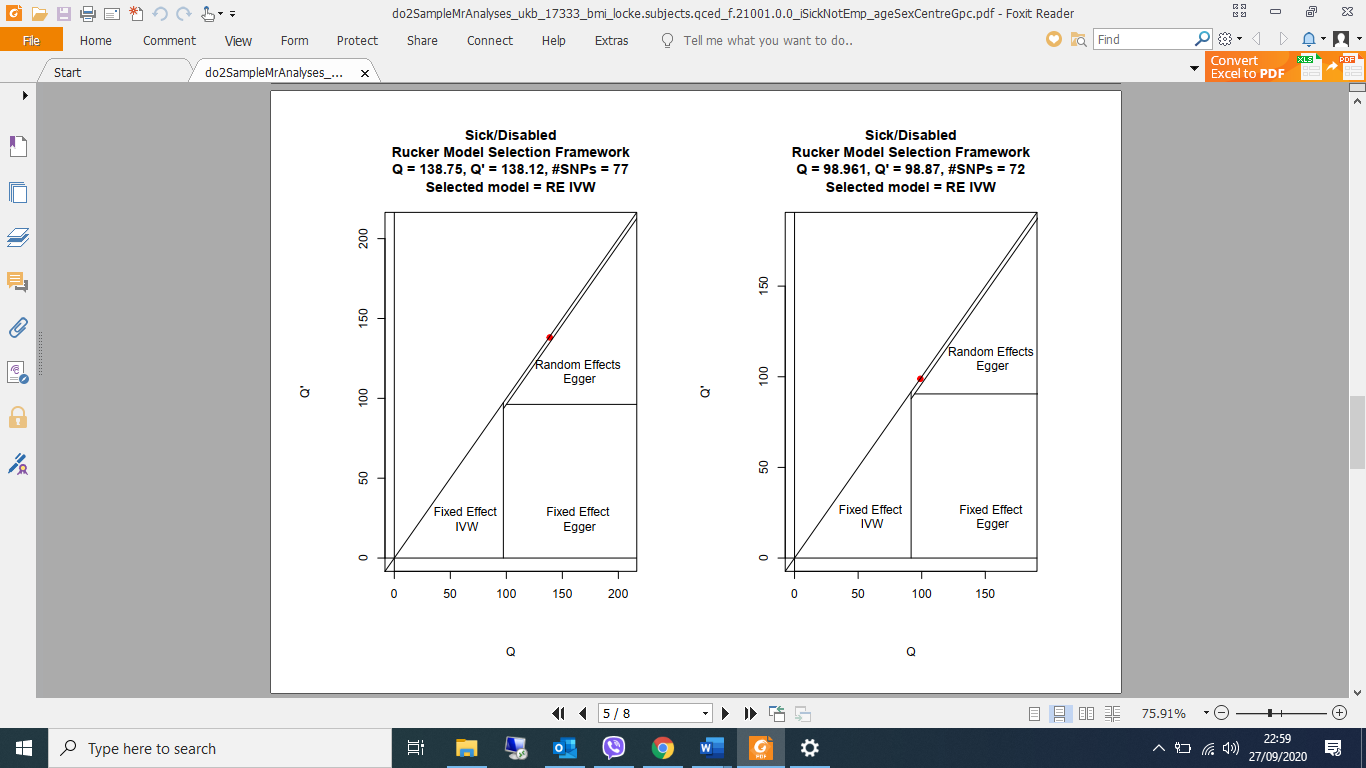


Footnote: The region in which the red dot lies indicates the model selected. The Random Effects IVW region has not been named in the diagram due to its small size. It is the narrow diagonal strip between the Random Effects Egger region and the main diagonal.

Figure S11 Galbraith Radial Plot for MR analysis for Sick/Disabled outcome


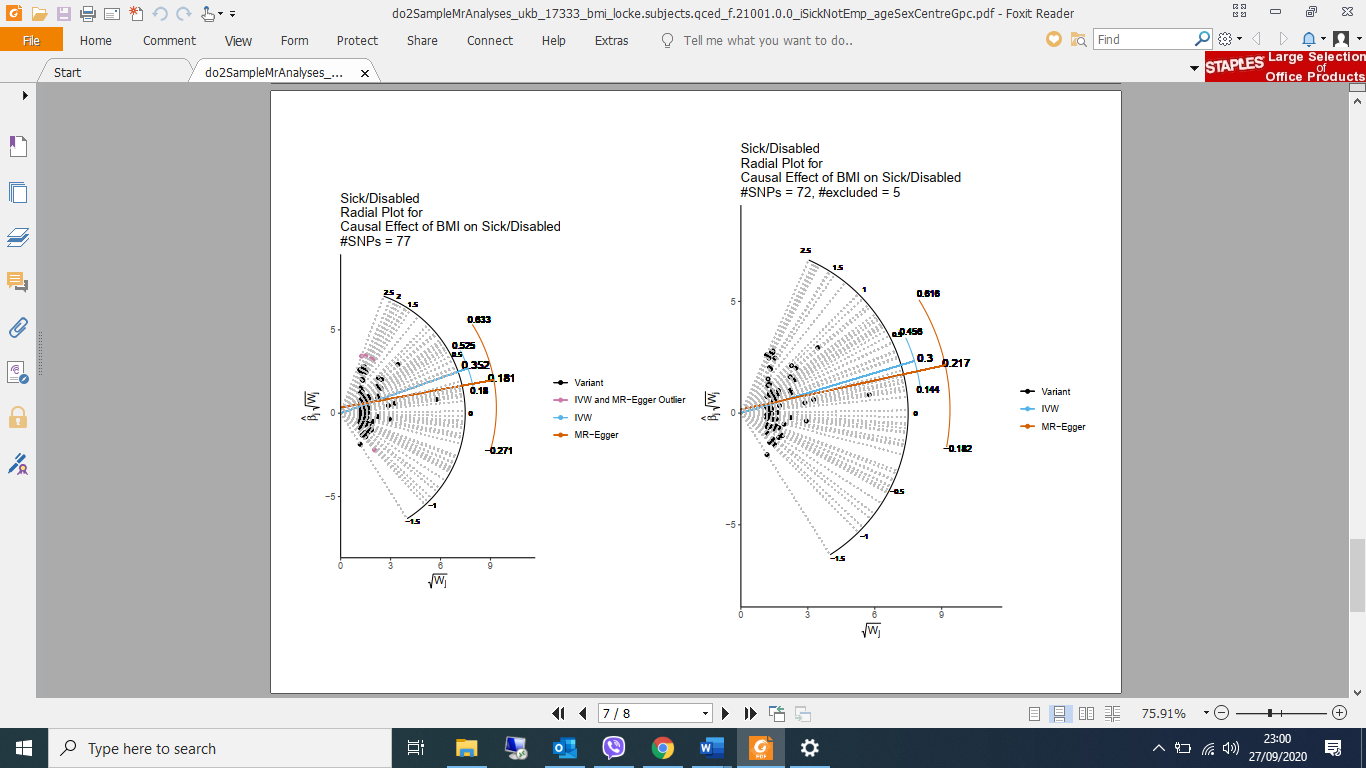


Footnote: $\sqrt{w_{j}}$ - square root of IVW weight given to SNP j. $\hat{\beta_{j}}$ – ratio causal effect estimate for SNP j. Pink Dot – Outlier SNP. Blue – IVW method fit plus 95% confidence interval. Orange – MR-Egger method fitted with its causal effect estimate plus 95% confidence interval.

Figure S12 Galbraith Radial Funnel Plot for MR analysis for Sick/Disabled outcome


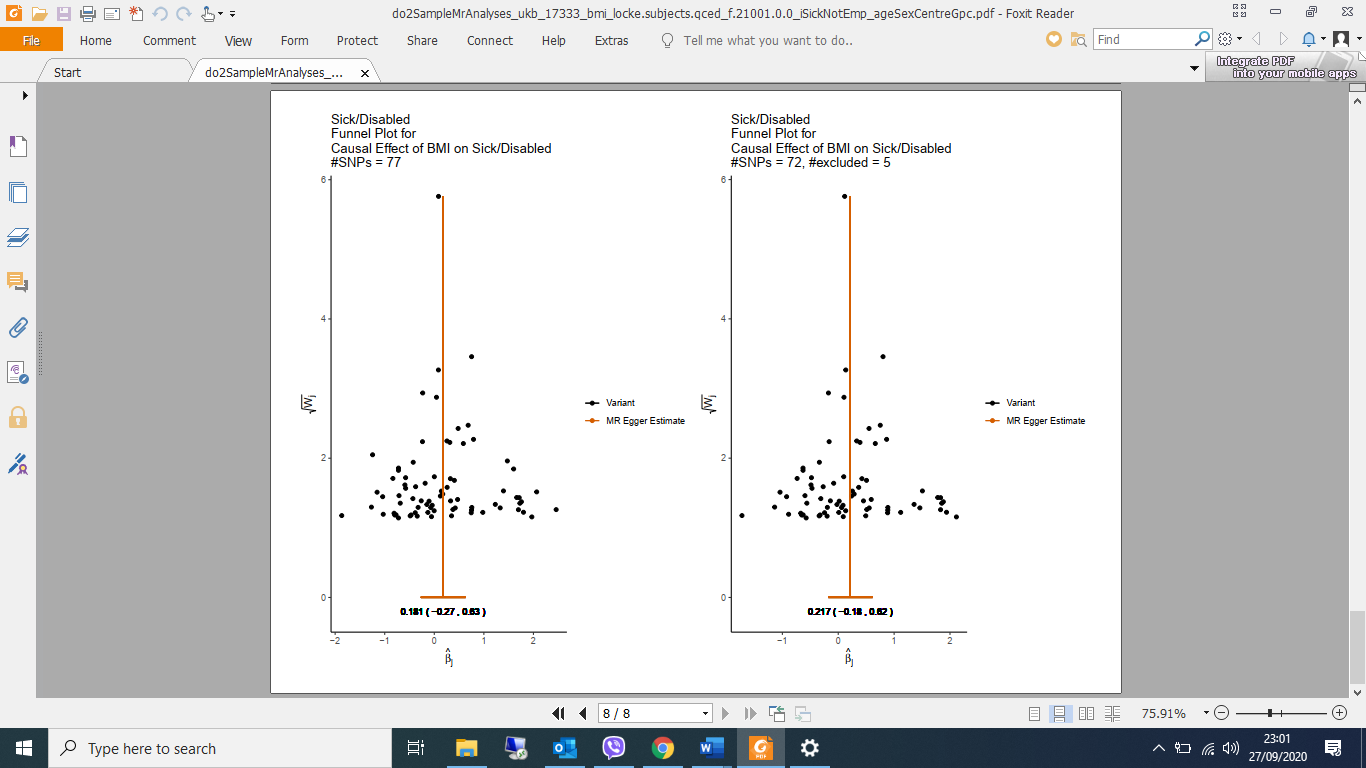


Footnote: $\sqrt{w_{j}}$ - square root of IVW weight given to SNP j. $\hat{\beta_{j}}$ – ratio causal effect estimate for SNP j. Blue – IVW method fit plus 95% confidence interval. Orange – MR-Egger method fitted with its causal effect estimate plus 95% confidence interval.

Figure S13 Scatter plots of Max Education Level-SNP associations versus exposure-SNP associations showing misfit of MR Egger Radial method. LHS – complete instrument SNP set. RHS after removal of outlier SNP.


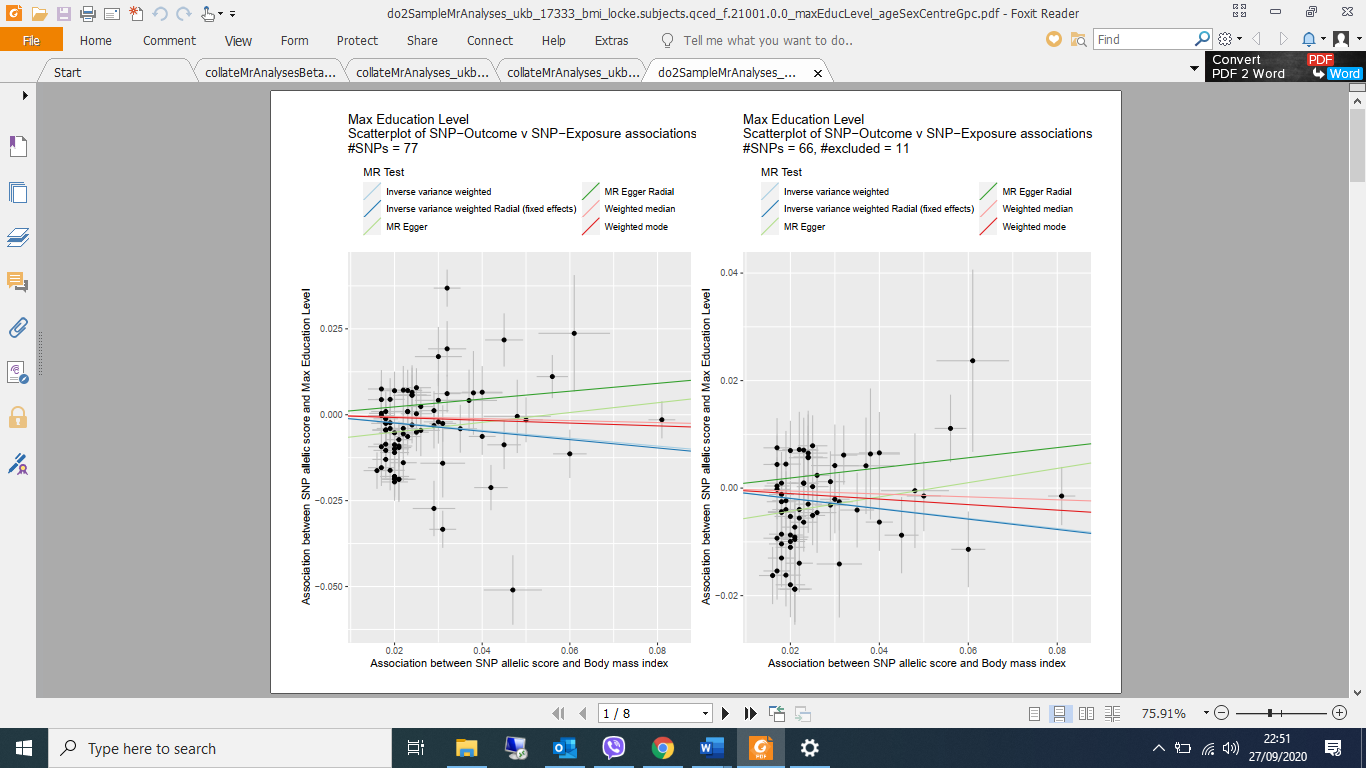


Scatter plot with x axis – BMI-SNP regression coefficient estimates from Locke et al. (normalised BMI), y axis – Max Education Level-SNP log odds from UK Biobank regressions. Also plotted are the fits for six causal effect estimation methods.

Figure S14 Comparison of actual versus predicted Sickness/Disability by BMI strata


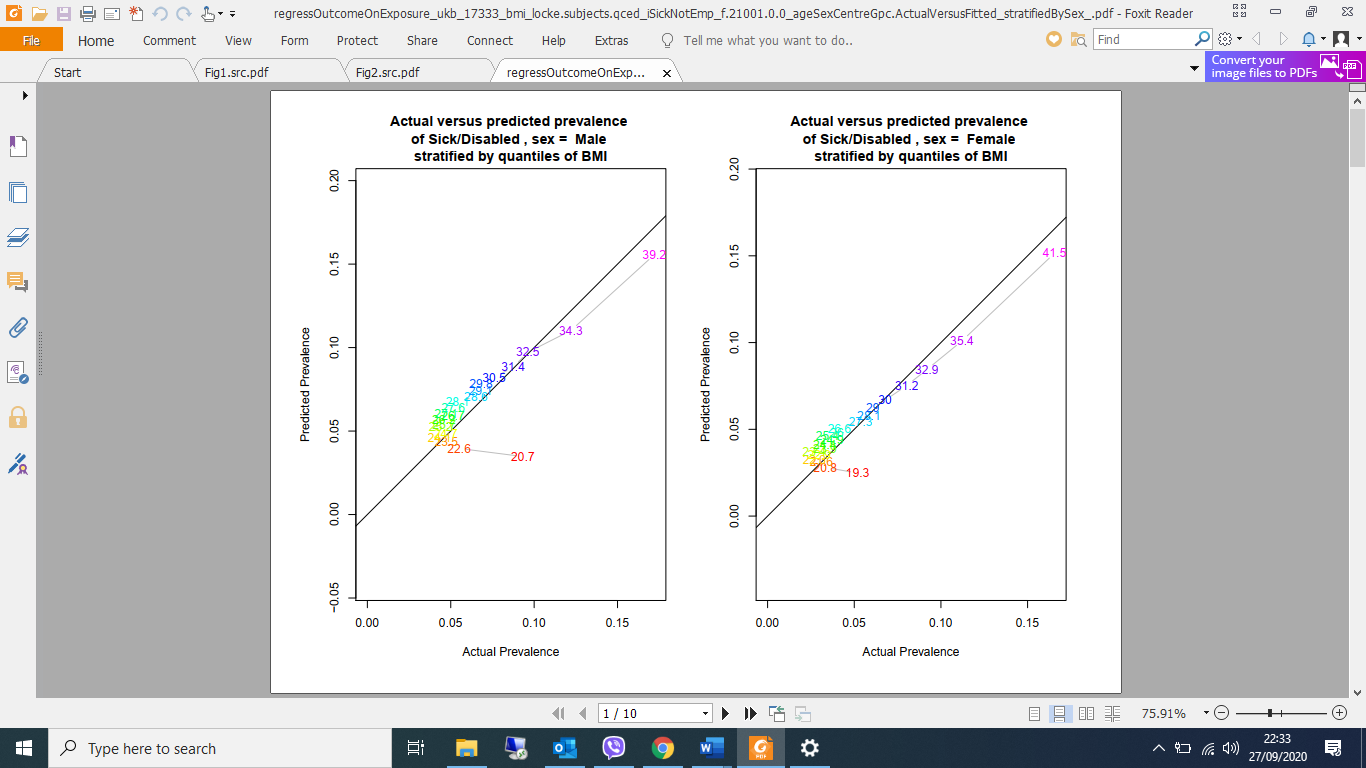


Footnote: Plotted coloured numbers indicate mean BMI in quantile.

# Tables

Table S1 Instrument SNP set used in the study

| **SNP** | **Chr** | **Effect**  **allele** | **Other**  **allele** | **gene** | **Beta** | **Std Err** | **P Value** | **Effect**  **allele**  **freq** |
| --- | --- | --- | --- | --- | --- | --- | --- | --- |
| rs11165643 | 1 | T | C | PTBP2 | 0.022 | 0.0031 | 1.0E-13 | 0.574 |
| rs12401738 | 1 | A | G | FUBP1 | 0.02 | 0.0031 | 2.0E-10 | 0.347 |
| rs12566985 | 1 | G | A | FPGT-TNNI3K | 0.024 | 0.0031 | 2.0E-15 | 0.455 |
| rs17024393 | 1 | C | T | GNAT2 | 0.061 | 0.0082 | 2.0E-13 | 0.043 |
| rs2820292 | 1 | C | A | NAV1 | 0.018 | 0.0031 | 5.0E-10 | 0.547 |
| rs3101336 | 1 | C | T | NEGR1 | 0.032 | 0.0031 | 6.0E-26 | 0.611 |
| rs543874 | 1 | G | A | SEC16B | 0.05 | 0.0038 | 2.0E-40 | 0.195 |
| rs657452 | 1 | A | G | AGBL4 | 0.023 | 0.0031 | 2.0E-13 | 0.397 |
| rs977747 | 1 | T | G | TAL1 | 0.017 | 0.0031 | 2.0E-08 | 0.403 |
| rs11191560 | 10 | C | T | NT5C2 | 0.031 | 0.0051 | 2.0E-09 | 0.091 |
| rs17094222 | 10 | C | T | HIF1AN | 0.025 | 0.0036 | 2.0E-11 | 0.209 |
| rs7899106 | 10 | G | A | GRID1 | 0.038 | 0.0066 | 1.0E-08 | 0.057 |
| rs7903146 | 10 | C | T | TCF7L2 | 0.024 | 0.0033 | 1.0E-12 | 0.713 |
| rs11030104 | 11 | A | G | BDNF | 0.042 | 0.0038 | 7.0E-30 | 0.791 |
| rs12286929 | 11 | G | A | CADM1 | 0.021 | 0.0031 | 5.0E-13 | 0.523 |
| rs2176598 | 11 | T | C | HSD17B12 | 0.019 | 0.0033 | 3.0E-08 | 0.256 |
| rs3817334 | 11 | T | C | MTCH2 | 0.026 | 0.0028 | 1.0E-17 | 0.401 |
| rs4256980 | 11 | G | C | TRIM66 | 0.021 | 0.0028 | 8.0E-12 | 0.638 |
| rs11057405 | 12 | G | A | CLIP1 | 0.03 | 0.0054 | 1.0E-08 | 0.902 |
| rs7138803 | 12 | A | G | BCDIN3D | 0.032 | 0.0031 | 5.0E-26 | 0.379 |
| rs12429545 | 13 | A | G | OLFM4 | 0.032 | 0.0043 | 3.0E-13 | 0.135 |
| rs1441264 | 13 | A | G | MIR548A2 | 0.017 | 0.0031 | 3.0E-08 | 0.613 |
| rs9540493 | 13 | A | G | MIR548X2 | 0.018 | 0.0031 | 4.0E-09 | 0.464 |
| rs10132280 | 14 | C | A | STXBP6 | 0.022 | 0.0033 | 1.0E-11 | 0.674 |
| rs12885454 | 14 | C | A | PRKD1 | 0.02 | 0.0031 | 9.0E-11 | 0.646 |
| rs7141420 | 14 | T | C | NRXN3 | 0.023 | 0.0028 | 9.0E-15 | 0.529 |
| rs16951275 | 15 | T | C | MAP2K5 | 0.03 | 0.0033 | 2.0E-18 | 0.771 |
| rs7164727 | 15 | T | C | LOC100287559 | 0.019 | 0.0031 | 4.0E-09 | 0.671 |
| rs12446632 | 16 | G | A | GPRC5B | 0.04 | 0.0046 | 2.0E-19 | 0.866 |
| rs1558902 | 16 | A | T | FTO | 0.081 | 0.0031 | 1.0E-156 | 0.409 |
| rs3888190 | 16 | A | C | ATP2A1 | 0.031 | 0.0031 | 3.0E-25 | 0.397 |
| rs758747 | 16 | T | C | NLRC3 | 0.023 | 0.0033 | 2.0E-10 | 0.28 |
| rs9925964 | 16 | A | G | KAT8 | 0.02 | 0.0031 | 9.0E-11 | 0.623 |
| rs1000940 | 17 | G | A | RABEP1 | 0.018 | 0.0033 | 2.0E-08 | 0.32 |
| rs12940622 | 17 | G | A | RPTOR | 0.018 | 0.0028 | 4.0E-10 | 0.572 |
| rs9914578 | 17 | G | C | SMG6 | 0.02 | 0.0036 | 2.0E-08 | 0.229 |
| rs1808579 | 18 | C | T | C18orf8 | 0.016 | 0.0031 | 4.0E-08 | 0.534 |
| rs6567160 | 18 | C | T | MC4R | 0.056 | 0.0036 | 7.0E-59 | 0.236 |
| rs7243357 | 18 | T | G | GRP | 0.022 | 0.0038 | 9.0E-09 | 0.813 |
| rs17724992 | 19 | A | G | PGPEP1 | 0.02 | 0.0033 | 8.0E-09 | 0.743 |
| rs2075650 | 19 | A | G | TOMM40 | 0.026 | 0.0043 | 3.0E-09 | 0.85 |
| rs2287019 | 19 | C | T | QPCTL | 0.035 | 0.0038 | 2.0E-18 | 0.806 |
| rs3810291 | 19 | A | G | ZC3H4 | 0.029 | 0.0033 | 6.0E-16 | 0.654 |
| rs1016287 | 2 | T | C | FLJ30838 | 0.023 | 0.0033 | 4.0E-12 | 0.285 |
| rs10182181 | 2 | G | A | ADCY3 | 0.031 | 0.0031 | 8.0E-26 | 0.468 |
| rs11126666 | 2 | A | G | KCNK3 | 0.02 | 0.0033 | 1.0E-09 | 0.283 |
| rs13021737 | 2 | G | A | TMEM18 | 0.06 | 0.0038 | 5.0E-54 | 0.83 |
| rs1528435 | 2 | T | C | UBE2E3 | 0.018 | 0.0028 | 5.0E-09 | 0.631 |
| rs17203016 | 2 | G | A | CREB1 | 0.021 | 0.0038 | 3.0E-08 | 0.195 |
| rs2121279 | 2 | T | C | LRP1B | 0.024 | 0.0043 | 2.0E-08 | 0.15 |
| rs7599312 | 2 | G | A | ERBB4 | 0.021 | 0.0033 | 5.0E-11 | 0.721 |
| rs6091540 | 20 | C | T | ZFP64 | 0.019 | 0.0033 | 2.0E-08 | 0.725 |
| rs2836754 | 21 | C | T | ETS2 | 0.017 | 0.0031 | 2.0E-08 | 0.599 |
| rs13078960 | 3 | G | T | CADM2 | 0.029 | 0.0036 | 1.0E-14 | 0.193 |
| rs1516725 | 3 | C | T | ETV5 | 0.045 | 0.0043 | 1.0E-24 | 0.869 |
| rs16851483 | 3 | T | G | RASA2 | 0.048 | 0.0077 | 2.0E-10 | 0.066 |
| rs2365389 | 3 | C | T | FHIT | 0.02 | 0.0028 | 1.0E-10 | 0.572 |
| rs3849570 | 3 | A | C | GBE1 | 0.018 | 0.0033 | 2.0E-08 | 0.362 |
| rs6804842 | 3 | G | A | RARB | 0.018 | 0.0031 | 8.0E-10 | 0.569 |
| rs10938397 | 4 | G | A | GNPDA2 | 0.04 | 0.0031 | 1.0E-40 | 0.428 |
| rs11727676 | 4 | T | C | HHIP | 0.037 | 0.0064 | 6.0E-09 | 0.911 |
| rs13107325 | 4 | T | C | SLC39A8 | 0.047 | 0.0066 | 1.0E-12 | 0.072 |
| rs17001654 | 4 | G | C | SCARB2 | 0.03 | 0.0054 | 5.0E-09 | 0.154 |
| rs2112347 | 5 | T | G | POC5 | 0.025 | 0.0028 | 2.0E-17 | 0.621 |
| rs7715256 | 5 | G | T | GALNT10 | 0.017 | 0.0028 | 9.0E-09 | 0.422 |
| rs13191362 | 6 | A | G | PARK2 | 0.029 | 0.0048 | 1.0E-09 | 0.88 |
| rs13201877 | 6 | G | A | IFNGR1 | 0.024 | 0.0043 | 4.0E-08 | 0.14 |
| rs205262 | 6 | G | A | C6orf106 | 0.021 | 0.0031 | 3.0E-10 | 0.285 |
| rs2207139 | 6 | G | A | TFAP2B | 0.045 | 0.0038 | 8.0E-31 | 0.176 |
| rs9374842 | 6 | T | C | LOC285762 | 0.02 | 0.0033 | 7.0E-09 | 0.748 |
| rs1167827 | 7 | G | A | HIP1 | 0.02 | 0.0031 | 2.0E-10 | 0.557 |
| rs17405819 | 8 | T | C | HNF4G | 0.022 | 0.0033 | 1.0E-11 | 0.702 |
| rs10733682 | 9 | A | G | LMX1B | 0.019 | 0.0031 | 2.0E-10 | 0.475 |
| rs10968576 | 9 | G | A | LINGO2 | 0.025 | 0.0033 | 2.0E-14 | 0.315 |
| rs1928295 | 9 | T | C | TLR4 | 0.018 | 0.0028 | 4.0E-10 | 0.55 |
| rs4740619 | 9 | T | C | C9orf93 | 0.017 | 0.0031 | 6.0E-09 | 0.54 |
| rs6477694 | 9 | C | T | EPB41L4B | 0.017 | 0.0031 | 2.0E-08 | 0.371 |

Table S2 UK Biobank Fields used in the study

| **UK Biobank field** | **Type** | **Description** | **Comment** |
| --- | --- | --- | --- |
|  |  |  |  |
| **QC Related** | | | |
| 21000 | categorical | Ethnic background | Amalgam of sequential branching questions asked during the initial Assessment Centre visit |
| 22006 | dichotomous | Genetic ethnic grouping | [Samples who self-identified as 'White British' according to Field 21000 and have very similar genetic ancestry based on a principal components analysis of the genotypes.](http://biobank.ctsu.ox.ac.uk/showcase/field.cgi?id=21000) |
| 22019 | dichotomous | Sex chromosome aneuploidy | Sex chromosome aneuploidy marker. This indicates samples which were identified as putatively carrying sex chromosome configurations that are not either XX or XY. |
| 22027 | dichotomous | Outliers for heterozygosity or missing rate | Indicates samples identified as outliers in heterozygosity and missing rates, which implies that the genotypes for these samples are of poor quality. |
| 22005 | continuous | Missingness | Missing rate of each sample based on a set of high-quality markers. |
|  |  |  |  |
| 22001 | dichotomous | Genetic sex | Sex as determined from genotyping analysis. |
| 31 | dichotomous | Sex | Sex of participant. Acquired from central registry at recruitment, but in some cases updated by the participant. |
|  |  |  |  |
| 22009 | continuous | Genetic principal components | Score for each principal component 1-40 |
|  |  |  |  |
| **Outcome Related** | | | |
| 6142 | categorical | self-reported current employment status |  |
| 767 | continuous | Length of working week for main job | Collected from all the participants who indicated they were in paid employment or self-employed (see Field 6142) |
| 189 | continuous | Townsend deprivation index at recruitment | Townsend deprivation index calculated immediately prior to participant joining UK Biobank. |
| 738 | ordinal | Average total household income before tax | Collected from participants except those who indicated they were living in a sheltered accommodation or in a care home |
| 6138 | ordinal | Education Qualifications |  |
|  |  |  |  |
| **Exposure Related** | | | |
| 21001 | continuous | Body mass index (BMI) | BMI value here is constructed from height and weight measured during the initial Assessment Centre visit. |

Table S3 Observational analyses for the association between BMI and employment related outcomes

| **Outcome** | **Regressor** | **Odds Ratio** | **Odds Ratio**  **95% CI** | **Odds Ratio**  **P Value** | **LRT P**  **Value** |
| --- | --- | --- | --- | --- | --- |
| Not in paid employment | Age | 1.150 | (1.148, 1.152) | 0 | 0.0E+00 |
| Not in paid employment | Assessment Centre | NA | NA | NA | 3.9E-119 |
| Not in paid employment | BMI | 1.015 | (1.013, 1.017) | 3.7E-43 | 1.0E-42 |
| Not in paid employment | Sex (=Female) | 1.130 | (1.105, 1.155) | 6.3E-27 | 5.9E-27 |
| Not in paid employment | PGC23 | 0.995 | (0.9913, 0.9984) | 5.0E-03 | 5.0E-03 |
| Sick/Disabled | BMI | 1.083 | (1.079, 1.087) | 0 | 0.0E+00 |
| Sick/Disabled | Age | 1.083 | (1.079, 1.086) | 0 | 0.0E+00 |
| Sick/Disabled | Assessment Centre | NA | NA | NA | 1.6E-218 |
| Sick/Disabled | PGC5 | 1.014 | (1.01, 1.019) | 1.1E-10 | 1.2E-10 |
| Sick/Disabled | PGC14 | 0.987 | (0.9801, 0.9931) | 6.1E-05 | 6.0E-05 |
| Caring for Home/Family | Sex (=Female) | 7.936 | (7.432, 8.474) | 0 | 0.0E+00 |
| Caring for Home/Family | Age | 1.020 | (1.016, 1.024) | 5.5E-24 | 3.5E-24 |
| Caring for Home/Family | Assessment Centre | NA | NA | NA | 1.9E-23 |
| Caring for Home/Family | BMI | 0.992 | (0.9874, 0.9963) | 3.4E-04 | 3.1E-04 |
| Caring for Home/Family | PGC26 | 0.991 | (0.9828, 0.9989) | 2.5E-02 | 2.5E-02 |
| Early Retirement | Age | 1.377 | (1.371, 1.383) | 0 | 0.0E+00 |
| Early Retirement | Assessment Centre | NA | NA | NA | 1.9E-60 |
| Early Retirement | PGC14 | 1.011 | (1.006, 1.016) | 9.0E-06 | 9.0E-06 |
| Early Retirement | BMI | 0.993 | (0.99, 0.9965) | 4.3E-05 | 4.1E-05 |
| Early Retirement | PGC10 | 1.012 | (1.004, 1.019) | 3.7E-03 | 3.7E-03 |
| Unemployed | Assessment Centre | NA | NA | NA | 2.6E-141 |
| Unemployed | Sex (=Female) | 0.547 | (0.5145, 0.5809) | 7.7E-85 | 4.3E-89 |
| Unemployed | Age | 1.023 | (1.018, 1.027) | 5.0E-24 | 2.6E-24 |
| Unemployed | BMI | 1.027 | (1.022, 1.033) | 1.9E-20 | 1.3E-19 |
| Unemployed | PGC14 | 0.985 | (0.9754, 0.9943) | 1.8E-03 | 1.7E-03 |
|  |  |  |  |  |  |
| **Outcome** | **Regressor** | **Beta** | **Beta**  **95% CI** | **Beta**  **P Value** | **LRT P**  **Value** |
| Townsend Deprivation Index | BMI | 0.0562 | (0.05381, 0.05856) | 0 | 0.0E+00 |
| Townsend Deprivation Index | Assessment Centre | NA | NA | NA | 0.0E+00 |
| Townsend Deprivation Index | Age | -0.0302 | (-0.03197, -0.02842) | 1.0E-242 | 8.7E-243 |
| Townsend Deprivation Index | PGC5 | 0.0250 | (0.02242, 0.02759) | 6.5E-80 | 6.2E-80 |
| Townsend Deprivation Index | PGC14 | -0.0237 | (-0.02761, -0.01972) | 6.1E-32 | 6.0E-32 |
| Hours Worked | Age | -0.2436 | (-0.2522, -0.235) | 0 | 0.0E+00 |
| Hours Worked | Sex (=Female) | -8.4085 | (-8.517, -8.3) | 0 | 0.0E+00 |
| Hours Worked | BMI | 0.1780 | (0.1666, 0.1895) | 2.3E-204 | 1.9E-204 |
| Hours Worked | Assessment Centre | NA | NA | NA | 4.2E-60 |
| Hours Worked | PGC5 | 0.0410 | (0.02888, 0.05303) | 3.0E-11 | 3.0E-11 |
|  |  |  |  |  |  |
| **Outcome** | **Regressor** | **Odds Ratio** | **Odds Ratio**  **95% CI** | **Odds Ratio**  **P Value** | **LRT P**  **Value** |
| Household Income | Age | 0.973 | (0.9715, 0.974) | 0 | 0.0E+00 |
| Household Income | Assessment Centre | NA | NA | NA | 0.0E+00 |
| Household Income | numberInHousehold Winsorised12 | 1.689 | (1.677, 1.701) | 0 | 0.0E+00 |
| Household Income | BMI | 0.974 | (0.9722, 0.9755) | 4.7E-214 | 3.3E-214 |
| Household Income | Sex (=Female) | 0.855 | (0.8412, 0.8692) | 2.2E-78 | 1.9E-78 |
| Max Education Level | BMI | 0.963 | (0.9616, 0.9648) | 0 | 0.0E+00 |
| Max Education Level | Assessment Centre | NA | NA | NA | 0.0E+00 |
| Max Education Level | Sex (=Female) | 0.846 | (0.8317, 0.8597) | 5.9E-88 | 5.3E-88 |
| Max Education Level | Age | 1.008 | (1.007, 1.009) | 5.7E-36 | 5.6E-36 |
| Max Education Level | PGC9 | 0.993 | (0.9907, 0.9948) | 4.0E-12 | 3.9E-12 |

Footnote: Results for the regression of outcomes on exposure. The table has been truncated to the 5 most significant regressors (according to LRT p-value) per outcome. Each outcome was regressed on age, sex, study assessment centre, and 40 genetic principal components (PGC). The Household Income outcome regression had an additional regressor - numberInHouseholdWinsorised12. LRT p-value – is the likelihood ratio test p-value for whether dropping the regressor from the model would worsen the fit. Regression estimates for sex are the effect of changing from Male to Female, *ceteris paribus*. Regression estimates are not given for Assessment Centre as there are multiple (one per assessment centre). Odds Ratio p-value – is the p-value for H0: Odds Ratio = 1. Beta p-value – is the p-value for H0: Regression Coefficient = 0.

Table S4 Regression of exposure on polygenic score

| **Regressor** | **Df** | **F value** | **Pr(>F)** | **Adj Partial**  **Rsq** | **Estimate** | **Std.**  **Error** | **t value** | **Pr(>\|t\|)** |
| --- | --- | --- | --- | --- | --- | --- | --- | --- |
| (Intercept) | NA | NA | NA | NA | 17.221 | 0.349 | 49.39 | <1.0E-300 |
| polyScore.nofAllele | 1 | 3708.7 | <1.0E-300 | 1.523% | 0.109 | 0.002 | 59.09 | <1.0E-300 |
| Sex | 1 | 1897.8 | <1.0E-300 | 0.799% | -0.884 | 0.021 | -42.39 | <1.0E-300 |
| Assessment Centre* | 21 | 84.9 | <1.0E-300 | 0.617% | NA | NA | NA | NA |
| Age | 1 | 1197.6 | 9.4E-262 | 0.212% | 0.034 | 0.001 | 22.87 | 1.1E-115 |
| PGC5 | 1 | 77.2 | 1.5E-18 | 0.023% | 0.017 | 0.002 | 7.37 | 1.7E-13 |
| PGC4 | 1 | 75.7 | 3.3E-18 | 0.005% | 0.017 | 0.005 | 3.45 | 5.6E-04 |
| PGC14 | 1 | 52.2 | 4.9E-13 | 0.020% | -0.024 | 0.003 | -6.91 | 4.9E-12 |
| PGC20 | 1 | 37.3 | 1.0E-09 | 0.018% | 0.023 | 0.004 | 6.59 | 4.3E-11 |
| PGC39 | 1 | 28.7 | 8.7E-08 | 0.012% | -0.019 | 0.004 | -5.36 | 8.1E-08 |
| PGC25 | 1 | 20.3 | 6.6E-06 | 0.008% | 0.016 | 0.004 | 4.39 | 1.2E-05 |

Footnote: Regression of exposure on polygenic score plus covariates. The left half of the table gives ANOVA results and adjusted partial R^2^. The right half gives regression coefficient estimates. The Sex regression coefficient using males as the reference group. The table is ordered by adjusted partial R squared. The table is truncated to the set of regressors with p < 1x10^-5^. PGC=Principal Genetic Component. *Assessment Centre was coded as a set of dummy variables and therefore the regression results are displayed as NA. Residual standard error: 4.734 on 228833 degrees of freedom. Multiple R-squared: 0.03588, Adjusted R-squared: 0.03561. F-statistic: 133.1 on 64 and 228833 DF, p-value: < 2.2e-16.

Table S5 Causal effect estimates (2sample MR) for BMI on employment status, before dropping outlier SNPs

| **Method** | **Log Odds Ratio**  **per 1StdDev**  **increase**  **in BMI** | **Log Odds Ratio**  **per 1StdDev**  **increase**  **in BMI StdErr** | **Odds Ratio**  **per 1Kgm2**  **increase**  **in BMI** | **Odds Ratio**  **95% CI** | **Odds Ratio**  **P Value** | **P Value**  **Less**  **Than**  **0.05** |
| --- | --- | --- | --- | --- | --- | --- |
|  |  |  |  |  |  |  |
| **Not in paid employment** |  |  |  |  |  |  |
| Maximum likelihood | 0.054 | 0.035 | 1.011 | (0.9971, 1.026) | 1.2E-01 |  |
| MR Egger | 0.016 | 0.095 | 1.003 | (0.9654, 1.043) | 8.6E-01 |  |
| MR Egger (bootstrap) | 0.040 | 0.081 | 1.008 | (0.9757, 1.042) | 3.1E-01 |  |
| Simple median | 0.075 | 0.056 | 1.016 | (0.9928, 1.039) | 1.8E-01 |  |
| Weighted median | 0.080 | 0.061 | 1.017 | (0.9918, 1.042) | 1.9E-01 |  |
| Penalised weighted median | 0.080 | 0.060 | 1.017 | (0.9922, 1.042) | 1.8E-01 |  |
| Inverse variance weighted | 0.054 | 0.039 | 1.011 | (0.9954, 1.027) | 1.6E-01 |  |
| IVW radial | 0.054 | 0.039 | 1.011 | (0.9954, 1.027) | 1.6E-01 |  |
| Inverse variance weighted  (multiplicative random effects) | 0.054 | 0.039 | 1.011 | (0.9954, 1.027) | 1.6E-01 |  |
| Inverse variance weighted  (fixed effects) | 0.054 | 0.034 | 1.011 | (0.9972, 1.025) | 1.2E-01 |  |
| Simple mode | 0.154 | 0.117 | 1.033 | (0.9847, 1.083) | 1.9E-01 |  |
| Weighted mode | 0.093 | 0.075 | 1.019 | (0.9886, 1.051) | 2.2E-01 |  |
| Weighted mode (NOME) | 0.093 | 0.075 | 1.019 | (0.9889, 1.051) | 2.2E-01 |  |
| Simple mode (NOME) | 0.154 | 0.118 | 1.033 | (0.9843, 1.083) | 1.9E-01 |  |
| Robust adjusted profile score (RAPS) | 0.053 | 0.042 | 1.011 | (0.994, 1.028) | 2.1E-01 |  |
| Sign concordance test | 0.117 | NA | 1.025 | NA | 3.6E-01 |  |
| Unweighted regression | 0.051 | 3.788 | 1.011 | (0.2164, 4.72) | 9.9E-01 |  |
| Inverse variance weighted Radial  (fixed effects) | 0.055 | 0.034 | 1.011 | (0.9974, 1.026) | 1.1E-01 |  |
| Inverse variance weighted Radial  (random effects) | 0.055 | 0.039 | 1.011 | (0.9953, 1.028) | 1.7E-01 |  |
| MR Egger Radial | 0.036 | 0.101 | 1.008 | (0.9669, 1.05) | 7.2E-01 |  |
|  |  |  |  |  |  |  |
| **Sick/Disabled** |  |  |  |  |  |  |
| Maximum likelihood | 0.357 | 0.065 | 1.077 | (1.049, 1.106) | 3.6E-08 | * |
| MR Egger | 0.006 | 0.209 | 1.001 | (0.9198, 1.09) | 9.8E-01 |  |
| MR Egger (bootstrap) | 0.247 | 0.150 | 1.053 | (0.9905, 1.119) | 5.0E-02 |  |
| Simple median | 0.208 | 0.109 | 1.044 | (0.9986, 1.092) | 5.8E-02 |  |
| Weighted median | 0.166 | 0.116 | 1.035 | (0.9874, 1.085) | 1.5E-01 |  |
| Penalised weighted median | 0.154 | 0.116 | 1.033 | (0.9852, 1.082) | 1.8E-01 |  |
| Inverse variance weighted | 0.352 | 0.087 | 1.076 | (1.039, 1.114) | 4.9E-05 | * |
| IVW radial | 0.352 | 0.087 | 1.076 | (1.039, 1.114) | 4.9E-05 | * |
| Inverse variance weighted  (multiplicative random effects) | 0.352 | 0.087 | 1.076 | (1.039, 1.114) | 4.9E-05 | * |
| Inverse variance weighted  (fixed effects) | 0.352 | 0.064 | 1.076 | (1.048, 1.104) | 3.7E-08 | * |
| Simple mode | 0.085 | 0.239 | 1.018 | (0.9235, 1.122) | 7.2E-01 |  |
| Weighted mode | 0.159 | 0.136 | 1.033 | (0.9777, 1.092) | 2.5E-01 |  |
| Weighted mode (NOME) | 0.171 | 0.140 | 1.036 | (0.9787, 1.097) | 2.3E-01 |  |
| Simple mode (NOME) | 0.085 | 0.240 | 1.018 | (0.9232, 1.122) | 7.2E-01 |  |
| Robust adjusted profile score (RAPS) | 0.312 | 0.097 | 1.067 | (1.026, 1.11) | 1.3E-03 | * |
| Sign concordance test | 0.247 | NA | 1.053 | NA | 4.0E-02 | * |
| Unweighted regression | 0.274 | 3.788 | 1.059 | (0.2267, 4.943) | 9.4E-01 |  |
| Inverse variance weighted Radial  (fixed effects) | 0.362 | 0.064 | 1.078 | (1.05, 1.107) | 1.7E-08 | * |
| Inverse variance weighted Radial  (random effects) | 0.358 | 0.085 | 1.077 | (1.041, 1.115) | 6.8E-05 | * |
| MR Egger Radial | 0.181 | 0.227 | 1.038 | (0.9467, 1.139) | 4.3E-01 |  |
|  |  |  |  |  |  |  |
| **Caring for Home/Family** |  |  |  |  |  |  |
| Maximum likelihood | -0.218 | 0.075 | 0.956 | (0.9272, 0.9852) | 3.5E-03 | * |
| MR Egger | -0.158 | 0.198 | 0.968 | (0.8927, 1.049) | 4.3E-01 |  |
| MR Egger (bootstrap) | -0.259 | 0.178 | 0.948 | (0.8814, 1.019) | 7.3E-02 |  |
| Simple median | -0.198 | 0.120 | 0.960 | (0.9141, 1.008) | 9.7E-02 |  |
| Weighted median | -0.262 | 0.124 | 0.947 | (0.9006, 0.996) | 3.5E-02 | * |
| Penalised weighted median | -0.262 | 0.124 | 0.947 | (0.9005, 0.9959) | 3.4E-02 | * |
| Inverse variance weighted | -0.217 | 0.081 | 0.956 | (0.9252, 0.9879) | 7.2E-03 | * |
| IVW radial | -0.217 | 0.081 | 0.956 | (0.9252, 0.9879) | 7.2E-03 | * |
| Inverse variance weighted  (multiplicative random effects) | -0.217 | 0.081 | 0.956 | (0.9252, 0.9879) | 7.2E-03 | * |
| Inverse variance weighted  (fixed effects) | -0.217 | 0.074 | 0.956 | (0.9277, 0.9852) | 3.4E-03 | * |
| Simple mode | -0.193 | 0.253 | 0.961 | (0.8669, 1.065) | 4.5E-01 |  |
| Weighted mode | -0.206 | 0.165 | 0.958 | (0.896, 1.025) | 2.1E-01 |  |
| Weighted mode (NOME) | -0.206 | 0.154 | 0.958 | (0.8999, 1.02) | 1.9E-01 |  |
| Simple mode (NOME) | -0.193 | 0.252 | 0.961 | (0.8671, 1.064) | 4.4E-01 |  |
| Robust adjusted profile score (RAPS) | -0.225 | 0.085 | 0.954 | (0.9222, 0.9879) | 7.9E-03 | * |
| Sign concordance test | -0.273 | NA | 0.945 | NA | 2.2E-02 | * |
| Unweighted regression | -0.201 | 3.788 | 0.959 | (0.2054, 4.479) | 9.6E-01 |  |
| Inverse variance weighted Radial  (fixed effects) | -0.221 | 0.074 | 0.955 | (0.9269, 0.9844) | 2.9E-03 | * |
| Inverse variance weighted Radial  (random effects) | -0.220 | 0.081 | 0.955 | (0.9244, 0.9874) | 8.2E-03 | * |
| MR Egger Radial | -0.187 | 0.212 | 0.962 | (0.8826, 1.049) | 3.8E-01 |  |
|  |  |  |  |  |  |  |
| **Retired** |  |  |  |  |  |  |
| Maximum likelihood | 0.038 | 0.048 | 1.008 | (0.9883, 1.028) | 4.3E-01 |  |
| MR Egger | 0.127 | 0.129 | 1.027 | (0.9741, 1.082) | 3.3E-01 |  |
| MR Egger (bootstrap) | 0.079 | 0.113 | 1.016 | (0.9708, 1.064) | 2.5E-01 |  |
| Simple median | -0.062 | 0.076 | 0.987 | (0.9574, 1.018) | 4.1E-01 |  |
| Weighted median | 0.046 | 0.077 | 1.010 | (0.9783, 1.042) | 5.5E-01 |  |
| Penalised weighted median | 0.047 | 0.080 | 1.010 | (0.9773, 1.043) | 5.6E-01 |  |
| Inverse variance weighted | 0.037 | 0.053 | 1.008 | (0.9863, 1.03) | 4.8E-01 |  |
| IVW radial | 0.037 | 0.053 | 1.008 | (0.9863, 1.03) | 4.8E-01 |  |
| Inverse variance weighted  (multiplicative random effects) | 0.037 | 0.053 | 1.008 | (0.9863, 1.03) | 4.8E-01 |  |
| Inverse variance weighted  (fixed effects) | 0.037 | 0.048 | 1.008 | (0.9883, 1.028) | 4.4E-01 |  |
| Simple mode | -0.144 | 0.179 | 0.971 | (0.9025, 1.044) | 4.2E-01 |  |
| Weighted mode | 0.056 | 0.111 | 1.012 | (0.967, 1.058) | 6.2E-01 |  |
| Weighted mode (NOME) | 0.056 | 0.118 | 1.012 | (0.9644, 1.061) | 6.4E-01 |  |
| Simple mode (NOME) | -0.144 | 0.177 | 0.971 | (0.9032, 1.043) | 4.2E-01 |  |
| Robust adjusted profile score (RAPS) | 0.031 | 0.054 | 1.007 | (0.9845, 1.029) | 5.7E-01 |  |
| Sign concordance test | -0.013 | NA | 0.997 | NA | 1.0E+00 |  |
| Unweighted regression | 0.093 | 3.788 | 1.020 | (0.2183, 4.761) | 9.8E-01 |  |
| Inverse variance weighted Radial  (fixed effects) | 0.038 | 0.048 | 1.008 | (0.9885, 1.028) | 4.3E-01 |  |
| Inverse variance weighted Radial  (random effects) | 0.038 | 0.054 | 1.008 | (0.9861, 1.03) | 4.8E-01 |  |
| MR Egger Radial | 0.090 | 0.138 | 1.019 | (0.9632, 1.078) | 5.2E-01 |  |
|  |  |  |  |  |  |  |
| **Unemployed** |  |  |  |  |  |  |
| Maximum likelihood | 0.017 | 0.094 | 1.004 | (0.9659, 1.043) | 8.6E-01 |  |
| MR Egger | 0.036 | 0.228 | 1.007 | (0.9182, 1.105) | 8.8E-01 |  |
| MR Egger (bootstrap) | 0.050 | 0.224 | 1.010 | (0.9225, 1.107) | 4.1E-01 |  |
| Simple median | -0.026 | 0.144 | 0.995 | (0.938, 1.055) | 8.6E-01 |  |
| Weighted median | 0.003 | 0.156 | 1.001 | (0.939, 1.066) | 9.8E-01 |  |
| Penalised weighted median | 0.007 | 0.157 | 1.001 | (0.9395, 1.067) | 9.7E-01 |  |
| Inverse variance weighted | 0.017 | 0.093 | 1.003 | (0.9661, 1.042) | 8.6E-01 |  |
| IVW radial | 0.017 | 0.090 | 1.003 | (0.9676, 1.041) | 8.5E-01 |  |
| Inverse variance weighted  (multiplicative random effects) | 0.017 | 0.090 | 1.003 | (0.9676, 1.041) | 8.5E-01 |  |
| Inverse variance weighted  (fixed effects) | 0.017 | 0.093 | 1.003 | (0.9661, 1.042) | 8.6E-01 |  |
| Simple mode | -0.060 | 0.303 | 0.988 | (0.8729, 1.117) | 8.4E-01 |  |
| Weighted mode | 0.030 | 0.170 | 1.006 | (0.9391, 1.078) | 8.6E-01 |  |
| Weighted mode (NOME) | 0.030 | 0.185 | 1.006 | (0.9333, 1.085) | 8.7E-01 |  |
| Simple mode (NOME) | -0.060 | 0.285 | 0.988 | (0.8795, 1.109) | 8.3E-01 |  |
| Sign concordance test | -0.065 | NA | 0.987 | NA | 6.5E-01 |  |
| Unweighted regression | -0.014 | 3.788 | 0.997 | (0.2135, 4.656) | 1.0E+00 |  |
| Inverse variance weighted Radial  (fixed effects) | 0.017 | 0.093 | 1.004 | (0.9661, 1.042) | 8.6E-01 |  |
| Inverse variance weighted Radial  (random effects) | 0.017 | 0.082 | 1.004 | (0.9705, 1.038) | 8.4E-01 |  |
| MR Egger Radial | 0.060 | 0.235 | 1.013 | (0.9202, 1.114) | 8.0E-01 |  |

Footnote: The causal effects are given above on the original scale of estimation (columns - Log Odds Ratio per 1 Std Dev increase in BMI, Log Odds Ratio per 1 Std Dev increase in BMI Std Err). They are also reported as Odds Ratio per 1Kg/m2 BMI plus 95% confidence interval (columns - Odds Ratio per 1 Kg/m2 increase in BMI, Odds Ratio 95% CI). The conversion was done using the standard deviation of BMI is our study sample, which was 4.816209. The ‘Sign concordance test’ method (as implemented in the TwoSampleMR package mr function) does not return a standard error.

Table S6 Causal effect estimates (2sample MR) for BMI on TDI and Weekly Hours Worked, before dropping outlier SNPs

| **Method** | **Beta**  **per 1StdDev**  **increase in**  **BMI** | **Beta**  **per 1StdDev**  **increase in**  **BMI StdErr** | **Beta**  **per 1Kgm2**  **increase in**  **BMI** | **Beta**  **95% CI** | **Beta**  **P Value** | **P Value**  **Less**  **Than**  **0.05** |
| --- | --- | --- | --- | --- | --- | --- |
|  |  |  |  |  |  |  |
| **Townsend Deprivation Index** |  |  |  |  |  |  |
| Maximum likelihood | 0.187 | 0.039 | 0.039 | (0.02305, 0.05441) | 1.3E-06 | * |
| MR Egger | 0.121 | 0.122 | 0.025 | (-0.02466, 0.07489) | 3.3E-01 |  |
| MR Egger (bootstrap) | 0.244 | 0.093 | 0.051 | (0.01297, 0.08824) | 1.0E-03 | * |
| Simple median | 0.128 | 0.067 | 0.026 | (-0.0006151, 0.05359) | 5.5E-02 |  |
| Weighted median | 0.117 | 0.070 | 0.024 | (-0.004385, 0.05293) | 9.7E-02 |  |
| Penalised weighted median | 0.108 | 0.077 | 0.022 | (-0.008821, 0.05347) | 1.6E-01 |  |
| Inverse variance weighted | 0.185 | 0.050 | 0.038 | (0.01807, 0.05862) | 2.1E-04 | * |
| IVW radial | 0.185 | 0.050 | 0.038 | (0.01808, 0.05863) | 2.1E-04 | * |
| Inverse variance weighted  (multiplicative random effects) | 0.185 | 0.050 | 0.038 | (0.01807, 0.05862) | 2.1E-04 | * |
| Inverse variance weighted  (fixed effects) | 0.185 | 0.038 | 0.038 | (0.02283, 0.05387) | 1.3E-06 | * |
| Simple mode | 0.411 | 0.183 | 0.085 | (0.01096, 0.1595) | 2.7E-02 | * |
| Weighted mode | 0.107 | 0.094 | 0.022 | (-0.01626, 0.06051) | 2.6E-01 |  |
| Weighted mode (NOME) | 0.107 | 0.095 | 0.022 | (-0.01647, 0.06073) | 2.6E-01 |  |
| Simple mode (NOME) | 0.411 | 0.179 | 0.085 | (0.01244, 0.158) | 2.4E-02 | * |
| Robust adjusted profile score (RAPS) | 0.170 | 0.058 | 0.035 | (0.01197, 0.05879) | 3.1E-03 | * |
| Sign concordance test | 0.195 | NA | 0.040 | NA | 1.1E-01 |  |
| Unweighted regression | 0.135 | 3.788 | 0.028 | (-1.513, 1.569) | 9.7E-01 |  |
| Inverse variance weighted Radial  (fixed effects) | 0.190 | 0.038 | 0.039 | (0.02379, 0.05491) | 7.2E-07 | * |
| Inverse variance weighted Radial  (random effects) | 0.188 | 0.052 | 0.039 | (0.01788, 0.05998) | 5.2E-04 | * |
| MR Egger Radial | 0.238 | 0.131 | 0.049 | (-0.003793, 0.1027) | 7.3E-02 |  |
|  |  |  |  |  |  |  |
| **Hours Worked** |  |  |  |  |  |  |
| Maximum likelihood | 0.215 | 0.180 | 0.045 | (-0.02836, 0.1177) | 2.3E-01 |  |
| MR Egger | 0.268 | 0.449 | 0.056 | (-0.1268, 0.2382) | 5.5E-01 |  |
| MR Egger (bootstrap) | 0.039 | 0.414 | 0.008 | (-0.1602, 0.1765) | 4.4E-01 |  |
| Simple median | 0.112 | 0.281 | 0.023 | (-0.09099, 0.1375) | 6.9E-01 |  |
| Weighted median | 0.056 | 0.278 | 0.012 | (-0.1014, 0.1248) | 8.4E-01 |  |
| Penalised weighted median | 0.045 | 0.290 | 0.009 | (-0.1084, 0.1272) | 8.8E-01 |  |
| Inverse variance weighted | 0.211 | 0.182 | 0.044 | (-0.03031, 0.118) | 2.5E-01 |  |
| IVW radial | 0.211 | 0.182 | 0.044 | (-0.03031, 0.118) | 2.5E-01 |  |
| Inverse variance weighted  (multiplicative random effects) | 0.211 | 0.182 | 0.044 | (-0.03031, 0.118) | 2.5E-01 |  |
| Inverse variance weighted  (fixed effects) | 0.211 | 0.178 | 0.044 | (-0.02867, 0.1164) | 2.4E-01 |  |
| Simple mode | 0.302 | 0.492 | 0.063 | (-0.1376, 0.2629) | 5.4E-01 |  |
| Weighted mode | 0.128 | 0.352 | 0.026 | (-0.1166, 0.1696) | 7.2E-01 |  |
| Weighted mode (NOME) | 0.128 | 0.340 | 0.026 | (-0.1118, 0.1647) | 7.1E-01 |  |
| Simple mode (NOME) | 0.302 | 0.540 | 0.063 | (-0.1568, 0.2822) | 5.8E-01 |  |
| Sign concordance test | 0.117 | NA | 0.024 | NA | 3.6E-01 |  |
| Unweighted regression | 0.258 | 3.788 | 0.054 | (-1.488, 1.595) | 9.5E-01 |  |
| Inverse variance weighted Radial  (fixed effects) | 0.214 | 0.178 | 0.045 | (-0.02801, 0.117) | 2.3E-01 |  |
| Inverse variance weighted Radial  (random effects) | 0.214 | 0.166 | 0.044 | (-0.02301, 0.112) | 2.0E-01 |  |
| MR Egger Radial | 0.231 | 0.479 | 0.048 | (-0.1472, 0.2429) | 6.3E-01 |  |

Footnote: The causal effects are given above on the original scale of estimation (columns - Beta per 1 Std Dev increase in BMI, Beta per 1 Std Dev increase in BMI Std Err). They are also reported as Beta per 1Kg/m2 BMI plus 95% confidence interval (columns - Beta per 1 Kg/m2 increase in BMI, Beta 95% CI). The conversion was done using the standard deviation of BMI is our study sample, which was 4.816209. The ‘Sign concordance test’ method (as implemented in the TwoSampleMR package mr function) does not return a standard error.

Table S7 Causal effect estimates (2sample MR) for BMI on Household Income Level and Max Education Level, before dropping outlier SNPs

| **Method** | **Log Odds Ratio**  **per 1StdDev**  **increase**  **in BMI** | **Log Odds Ratio**  **per 1StdDev**  **increase**  **in BMI StdErr** | **Odds Ratio**  **per 1Kgm2**  **increase**  **in BMI** | **Odds Ratio**  **95% CI** | **Odds Ratio**  **P Value** | **P Value**  **Less**  **Than**  **0.05** |
| --- | --- | --- | --- | --- | --- | --- |
|  |  |  |  |  |  |  |
| **Highest Educational Attainment** |  |  |  |  |  |  |
| Maximum likelihood | -0.118 | 0.025 | 0.976 | (0.9657, 0.9858) | 3.0E-06 | * |
| MR Egger | 0.142 | 0.113 | 1.030 | (0.9836, 1.079) | 2.1E-01 |  |
| MR Egger (bootstrap) | 0.047 | 0.063 | 1.010 | (0.9842, 1.036) | 2.3E-01 |  |
| Simple median | -0.124 | 0.043 | 0.975 | (0.9575, 0.992) | 4.4E-03 | * |
| Weighted median | -0.029 | 0.045 | 0.994 | (0.976, 1.012) | 5.3E-01 |  |
| Penalised weighted median | -0.025 | 0.047 | 0.995 | (0.9759, 1.014) | 6.0E-01 |  |
| Inverse variance weighted | -0.114 | 0.048 | 0.977 | (0.9578, 0.9958) | 1.7E-02 | * |
| IVW radial | -0.114 | 0.048 | 0.977 | (0.9578, 0.9958) | 1.7E-02 | * |
| Inverse variance weighted  (multiplicative random effects) | -0.114 | 0.048 | 0.977 | (0.9578, 0.9958) | 1.7E-02 | * |
| Inverse variance weighted  (fixed effects) | -0.114 | 0.025 | 0.977 | (0.9669, 0.9865) | 3.8E-06 | * |
| Simple mode | -0.060 | 0.086 | 0.988 | (0.9539, 1.023) | 4.9E-01 |  |
| Weighted mode | -0.041 | 0.048 | 0.992 | (0.9725, 1.011) | 4.0E-01 |  |
| Weighted mode (NOME) | -0.041 | 0.052 | 0.992 | (0.9709, 1.013) | 4.4E-01 |  |
| Simple mode (NOME) | -0.060 | 0.087 | 0.988 | (0.9533, 1.023) | 4.9E-01 |  |
| Robust adjusted profile score (RAPS) | -0.101 | 0.051 | 0.979 | (0.9591, 1) | 5.0E-02 | * |
| Sign concordance test | -0.273 | NA | 0.945 | NA | 2.2E-02 | * |
| Unweighted regression | -0.086 | 3.788 | 0.982 | (0.2103, 4.587) | 9.8E-01 |  |
| Inverse variance weighted Radial  (fixed effects) | -0.121 | 0.025 | 0.975 | (0.9655, 0.9851) | 1.0E-06 | * |
| Inverse variance weighted Radial  (random effects) | -0.116 | 0.053 | 0.976 | (0.9553, 0.9976) | 3.3E-02 | * |
| MR Egger Radial | 0.114 | 0.123 | 1.024 | (0.9741, 1.076) | 3.6E-01 |  |
|  |  |  |  |  |  |  |
| **Household Income** |  |  |  |  |  |  |
| Maximum likelihood | -0.116 | 0.026 | 0.976 | (0.9658, 0.9868) | 1.2E-05 | * |
| MR Egger | -0.089 | 0.087 | 0.982 | (0.9476, 1.017) | 3.1E-01 |  |
| MR Egger (bootstrap) | -0.105 | 0.065 | 0.978 | (0.953, 1.005) | 5.5E-02 |  |
| Simple median | -0.053 | 0.043 | 0.989 | (0.9718, 1.007) | 2.2E-01 |  |
| Weighted median | -0.063 | 0.046 | 0.987 | (0.9685, 1.006) | 1.7E-01 |  |
| Penalised weighted median | -0.062 | 0.046 | 0.987 | (0.9688, 1.006) | 1.8E-01 |  |
| Inverse variance weighted | -0.116 | 0.035 | 0.976 | (0.9622, 0.9903) | 9.9E-04 | * |
| IVW radial | -0.116 | 0.035 | 0.976 | (0.9622, 0.9903) | 9.9E-04 | * |
| Inverse variance weighted  (multiplicative random effects) | -0.116 | 0.035 | 0.976 | (0.9622, 0.9903) | 9.9E-04 | * |
| Inverse variance weighted  (fixed effects) | -0.116 | 0.026 | 0.976 | (0.9658, 0.9866) | 8.4E-06 | * |
| Simple mode | -0.014 | 0.098 | 0.997 | (0.9583, 1.038) | 8.9E-01 |  |
| Weighted mode | -0.066 | 0.052 | 0.986 | (0.9657, 1.008) | 2.1E-01 |  |
| Weighted mode (NOME) | -0.071 | 0.057 | 0.985 | (0.9628, 1.008) | 2.1E-01 |  |
| Simple mode (NOME) | -0.014 | 0.098 | 0.997 | (0.9583, 1.038) | 8.9E-01 |  |
| Robust adjusted profile score (RAPS) | -0.106 | 0.037 | 0.978 | (0.9634, 0.9931) | 4.3E-03 | * |
| Sign concordance test | -0.247 | NA | 0.950 | NA | 4.0E-02 | * |
| Unweighted regression | -0.108 | 3.788 | 0.978 | (0.2094, 4.566) | 9.8E-01 |  |
| Inverse variance weighted Radial  (fixed effects) | -0.120 | 0.026 | 0.975 | (0.9651, 0.9859) | 4.9E-06 | * |
| Inverse variance weighted Radial  (random effects) | -0.118 | 0.036 | 0.976 | (0.9614, 0.9903) | 1.8E-03 | * |
| MR Egger Radial | -0.105 | 0.093 | 0.978 | (0.942, 1.016) | 2.6E-01 |  |

Footnote: The causal effects are given above on the original scale of estimation (columns - Beta per 1 Std Dev increase in BMI, Beta per 1 Std Dev increase in BMI Std Err). They are also reported as Beta per 1Kg/m2 BMI plus 95% confidence interval (columns - Beta per 1 Kg/m2 increase in BMI, Beta 95% CI). The conversion was done using the standard deviation of BMI is our study sample, which was 4.816209. The ‘Sign concordance test’ method (as implemented in the TwoSampleMR package mr function) does not return a standard error.

Table S8 Heterogeneity tests

|  |  | **Including outlier SNPs** | | |  | **Excluding outlier SNPs** | | |  |
| --- | --- | --- | --- | --- | --- | --- | --- | --- | --- |
| **Outcome** | **Method** | **Q** | **Q df** | **Q**  **P Value** | **P Value**  **Less**  **Than**  **0.05** | **Q** | **Q df** | **Q**  **P Value** | **P Value**  **Less**  **Than**  **0.05** |
| Not in paid employment | Inverse variance weighted | 96.25 | 76 | 5.8E-02 |  | 77.99 | 74 | 3.5E-01 |  |
| Not in paid employment | IVW radial | 96.21 | 76 | 5.9E-02 |  | 77.96 | 74 | 3.5E-01 |  |
| Not in paid employment | Maximum likelihood | 96.22 | 76 | 5.9E-02 |  | 77.97 | 74 | 3.5E-01 |  |
| Not in paid employment | MR Egger | 96.02 | 75 | 5.1E-02 |  | 77.66 | 73 | 3.3E-01 |  |
| Not in paid employment | Unweighted regression | 0.01 | 76 | 1.0E+00 |  | 0.01 | 74 | 1.0E+00 |  |
| Sick/Disabled | Inverse variance weighted | 139.58 | 76 | 1.2E-05 | * | 99.37 | 71 | 1.5E-02 | * |
| Sick/Disabled | IVW radial | 138.75 | 76 | 1.5E-05 | * | 98.96 | 71 | 1.6E-02 | * |
| Sick/Disabled | Maximum likelihood | 138.99 | 76 | 1.4E-05 | * | 99.02 | 71 | 1.6E-02 | * |
| Sick/Disabled | MR Egger | 133.70 | 75 | 3.6E-05 | * | 95.91 | 70 | 2.2E-02 | * |
| Sick/Disabled | Unweighted regression | 0.04 | 76 | 1.0E+00 |  | 0.03 | 71 | 1.0E+00 |  |
| Caring for Home/Family | Inverse variance weighted | 90.38 | 76 | 1.2E-01 |  | 71.22 | 74 | 5.7E-01 |  |
| Caring for Home/Family | IVW radial | 90.22 | 76 | 1.3E-01 |  | 71.09 | 74 | 5.7E-01 |  |
| Caring for Home/Family | Maximum likelihood | 90.25 | 76 | 1.3E-01 |  | 71.10 | 74 | 5.7E-01 |  |
| Caring for Home/Family | MR Egger | 90.25 | 75 | 1.1E-01 |  | 71.09 | 73 | 5.4E-01 |  |
| Caring for Home/Family | Unweighted regression | 0.04 | 76 | 1.0E+00 |  | 0.03 | 74 | 1.0E+00 |  |
| Retired | Inverse variance weighted | 92.59 | 76 | 9.5E-02 |  | 85.35 | 75 | 1.9E-01 |  |
| Retired | IVW radial | 92.58 | 76 | 9.5E-02 |  | 85.35 | 75 | 1.9E-01 |  |
| Retired | Maximum likelihood | 92.58 | 76 | 9.5E-02 |  | 85.35 | 75 | 1.9E-01 |  |
| Retired | MR Egger | 91.88 | 75 | 9.0E-02 |  | 85.02 | 74 | 1.8E-01 |  |
| Retired | Unweighted regression | 0.02 | 76 | 1.0E+00 |  | 0.01 | 75 | 1.0E+00 |  |
| Unemployed | Inverse variance weighted | 69.94 | 76 | 6.7E-01 |  | 69.94 | 76 | 6.7E-01 |  |
| Unemployed | IVW radial | 69.94 | 76 | 6.7E-01 |  | 69.94 | 76 | 6.7E-01 |  |
| Unemployed | Maximum likelihood | 69.94 | 76 | 6.7E-01 |  | 69.94 | 76 | 6.7E-01 |  |
| Unemployed | MR Egger | 69.93 | 75 | 6.4E-01 |  | 69.93 | 75 | 6.4E-01 |  |
| Unemployed | Unweighted regression | 0.04 | 76 | 1.0E+00 |  | 0.04 | 76 | 1.0E+00 |  |
| Hours Worked | Inverse variance weighted | 79.49 | 76 | 3.7E-01 |  | 63.09 | 74 | 8.1E-01 |  |
| Hours Worked | IVW radial | 79.47 | 76 | 3.7E-01 |  | 63.07 | 74 | 8.1E-01 |  |
| Hours Worked | Maximum likelihood | 79.47 | 76 | 3.7E-01 |  | 63.07 | 74 | 8.1E-01 |  |
| Hours Worked | MR Egger | 79.47 | 75 | 3.4E-01 |  | 63.08 | 73 | 7.9E-01 |  |
| Hours Worked | Unweighted regression | 0.17 | 76 | 1.0E+00 |  | 0.14 | 74 | 1.0E+00 |  |
| Townsend Deprivation Index | Inverse variance weighted | 129.63 | 76 | 1.2E-04 | * | 118.59 | 75 | 1.0E-03 | * |
| Townsend Deprivation Index | IVW radial | 129.04 | 76 | 1.4E-04 | * | 118.13 | 75 | 1.1E-03 | * |
| Townsend Deprivation Index | Maximum likelihood | 129.27 | 76 | 1.3E-04 | * | 118.30 | 75 | 1.1E-03 | * |
| Townsend Deprivation Index | MR Egger | 129.07 | 75 | 1.1E-04 | * | 118.32 | 74 | 8.2E-04 | * |
| Townsend Deprivation Index | Unweighted regression | 0.01 | 76 | 1.0E+00 |  | 0.01 | 75 | 1.0E+00 |  |
| Max Education Level | Inverse variance weighted | 287.15 | 76 | 2.8E-26 | * | 111.46 | 65 | 3.0E-04 | * |
| Max Education Level | IVW radial | 285.94 | 76 | 4.4E-26 | * | 111.16 | 65 | 3.2E-04 | * |
| Max Education Level | Maximum likelihood | 286.21 | 76 | 4.0E-26 | * | 111.23 | 65 | 3.1E-04 | * |
| Max Education Level | MR Egger | 265.43 | 75 | 4.3E-23 | * | 95.77 | 64 | 6.2E-03 | * |
| Max Education Level | Unweighted regression | 0.01 | 76 | 1.0E+00 |  | 0.00 | 65 | 1.0E+00 |  |
| Household Income | Inverse variance weighted | 139.11 | 76 | 1.4E-05 | * | 83.74 | 70 | 1.3E-01 |  |
| Household Income | IVW radial | 138.58 | 76 | 1.6E-05 | * | 83.55 | 70 | 1.3E-01 |  |
| Household Income | Maximum likelihood | 138.72 | 76 | 1.5E-05 | * | 83.62 | 70 | 1.3E-01 |  |
| Household Income | MR Egger | 138.89 | 75 | 1.0E-05 | * | 83.62 | 69 | 1.1E-01 |  |
| Household Income | Unweighted regression | 0.01 | 76 | 1.0E+00 |  | 0.00 | 70 | 1.0E+00 |  |

Footnote: Heterogeneity test results. Q – test statistic, Q df – degrees of freedom, P-value – p-value for H0 no heterogeneity across SNPs in causal effect size estimate.

Table S9 Unbalanced pleiotropy tests

|  |  | **Including outlier SNPs** | |  |  | **Excluding outlier SNPs** | |  |  |
| --- | --- | --- | --- | --- | --- | --- | --- | --- | --- |
| **Outcome** | **Method** | **Egger**  **intercept** | **Std**  **Err** | **P Value** | **P Value**  **Less**  **Than**  **0.05** | **Egger**  **intercept** | **Std**  **Err** | **P Value** | **P Value**  **Less**  **Than**  **0.05** |
| Not in paid employment | MR Egger | 0.0011 | 0.0027 | 6.7E-01 |  | 0.0014 | 0.0024 | 5.8E-01 |  |
| Sick/Disabled | MR Egger | 0.0107 | 0.0059 | 7.4E-02 |  | 0.0083 | 0.0052 | 1.2E-01 |  |
| Caring for Home/Family | MR Egger | -0.0018 | 0.0056 | 7.5E-01 |  | 0.0018 | 0.0052 | 7.2E-01 |  |
| Retired | MR Egger | -0.0028 | 0.0036 | 4.5E-01 |  | -0.0019 | 0.0035 | 5.9E-01 |  |
| Unemployed | MR Egger | -0.0006 | 0.0064 | 9.3E-01 |  | -0.0006 | 0.0064 | 9.3E-01 |  |
| Hours Worked | MR Egger | -0.0018 | 0.0126 | 8.9E-01 |  | -0.0007 | 0.0124 | 9.5E-01 |  |
| Townsend Deprivation Index | MR Egger | 0.0020 | 0.0034 | 5.7E-01 |  | 0.0014 | 0.0033 | 6.8E-01 |  |
| Max Education Level | MR Egger | -0.0079 | 0.0032 | 1.5E-02 | * | -0.0069 | 0.0021 | 1.9E-03 | * |
| Household Income | MR Egger | -0.0009 | 0.0024 | 7.3E-01 |  | 0.0007 | 0.0020 | 7.5E-01 |  |

Table S10 Rücker Model Selection Framework

|  | **Including outlier SNPs** | |  |  | **Excluding outlier SNPs** | |  |  |
| --- | --- | --- | --- | --- | --- | --- | --- | --- |
| **Outcome** | **Cochrans**  **Q** | **Ruckers**  **Q** | **# SNPs** | **Selected Model** | **Cochrans**  **Q** | **Ruckers**  **Q** | **# SNPs** | **Selected Model** |
| Not in paid employment | 96.21 | 96.19 | 77 | Inverse variance weighted  (fixed effects) | 77.96 | 77.96 | 75 | Inverse variance weighted  (fixed effects) |
| Sick/Disabled | 138.75 | 138.12 | 77 | Inverse variance weighted  (multiplicative random effects) | 98.96 | 98.87 | 72 | Inverse variance weighted  (multiplicative random effects) |
| Caring for Home/Family | 90.22 | 90.23 | 77 | Inverse variance weighted  (fixed effects) | 71.09 | 71.06 | 75 | Inverse variance weighted  (fixed effects) |
| Retired | 92.58 | 92.31 | 77 | Inverse variance weighted  (fixed effects) | 85.35 | 84.82 | 76 | Inverse variance weighted  (fixed effects) |
| Unemployed | 69.94 | 69.90 | 77 | Inverse variance weighted  (fixed effects) | 69.94 | 69.90 | 77 | Inverse variance weighted  (fixed effects) |
|  |  |  |  |  |  |  |  |  |
| Townsend Deprivation Index | 129.04 | 128.32 | 77 | Inverse variance weighted  (multiplicative random effects) | 118.13 | 117.26 | 76 | Inverse variance weighted  (multiplicative random effects) |
| Hours Worked | 79.47 | 79.47 | 77 | Inverse variance weighted  (fixed effects) | 63.07 | 63.06 | 75 | Inverse variance weighted  (fixed effects) |
|  |  |  |  |  |  |  |  |  |
| Max Education Level | 285.94 | 271.30 | 77 | RE Egger | 111.16 | 101.62 | 66 | RE Egger |
| Household Income | 138.58 | 138.64 | 77 | Inverse variance weighted  (multiplicative random effects) | 83.55 | 82.60 | 71 | Inverse variance weighted  (fixed effects) |

Footnote: The less parsimonious model was rejected at a p-value of > 0.05.

Table S11 Demographics summary for household income observed and missing strata

| **Household Income** | **observed** | **missing** |
| --- | --- | --- |
|  |  |  |
| Sample size (%) | 206457 (89.9) | 23116 (10.1) |
| sex = Female (%) | 92296 (44.7) | 12072 (52.2) |
| age (mean (SD)) | 52.74 (6.66) | 53.91 (6.36) |
| BMI (mean (SD)) | 27.36 (4.79) | 27.62 (5.11) |
|  |  |  |
| Employment Category (%) |  |  |
| Employed | 159506 (77.3) | 14595 (63.1) |
| Early Retirement | 26991 (13.1) | 3945 (17.1) |
| Sick/Disabled | 9590 (4.6) | 2242 (9.7) |
| Caring for Home/Family | 7070 (3.4) | 1568 (6.8) |
| Unemployed | 4327 (2.1) | 887 (3.8) |
| Not in paid Employment | 46951 (22.7) | 8521 (36.9) |
|  |  |  |
| Highest Educational Attainment (%) |  |  |
| None of the below | 20863 (10.1) | 5178 (22.4) |
| CSEs or equivalent | 9272 (4.5) | 1466 (6.3) |
| O levels/GCSEs or equivalent | 25009 (12.1) | 3271 (14.2) |
| A levels/AS levels or equivalent | 12260 (5.9) | 1118 (4.8) |
| NVQ or HND or HNC or equivalent | 31066 (15.0) | 3558 (15.4) |
| Other professional qualifications eg: nursing, teaching | 30387 (14.7) | 3303 (14.3) |
| College or University degree | 77044 (37.3) | 4312 (18.7) |
| NA | 556 (0.3) | 910 (3.9) |
|  |  |  |
| Hours Worked Weekly (mean (SD)) | 36.69 (11.92) | 34.14 (12.91) |
| Townsend Deprivation Index (mean (SD)) | -1.51 (2.96) | -1.33 (3.12) |

# References

1. Locke AE, Kahali B, Berndt SI, Justice AE, Pers TH, Day FR *et al.* Genetic studies of body mass index yield new insights for obesity biology. *Nature* 2015; **518:** 197.

2. Hemani G, Zheng J, Elsworth B, Wade KH, Haberland V, Baird D *et al.* The MR-Base platform supports systematic causal inference across the human phenome. *eLife* 2018; **7:** e34408.

3. Chang CC, Chow CC, Tellier LC, Vattikuti S, Purcell SM, Lee JJ. Second-generation PLINK: rising to the challenge of larger and richer datasets. *GigaScience* 2015; **4**(1)**:** 7.

4. Bowden J, Spiller W, Del Greco M F, Sheehan N, Thompson J, Minelli C *et al.* Improving the visualization, interpretation and analysis of two-sample summary data Mendelian randomization via the Radial plot and Radial regression. *International Journal of Epidemiology* 2018**:** dyy101-dyy101.

5. Rücker G, Schwarzer G, Carpenter JR, Binder H, Schumacher M. Treatment-effect estimates adjusted for small-study effects via a limit meta-analysis. *Biostatistics* 2010; **12**(1)**:** 122-142.
